# Nuclear–Electronic Orbital Coupled Cluster Theory with the Nuclear Hartree Product Representation for Multiple Quantum Nuclei and Application to Geometric Isotope Effects

**DOI:** 10.1021/acs.jctc.6c01568

**Published:** 2026-10-07

**Authors:** Rowan J. Goudy, Sharon Hammes-Schiffer

**Affiliations:** Department of Chemistry, 6740 Princeton University, Princeton, New Jersey 08544, United States

## Abstract

Nuclear–electronic orbital (NEO) methods treat specified nuclei, typically protons, quantum mechanically on the same level as electrons within quantum chemical calculations. This approach allows the computationally efficient inclusion of nuclear quantum effects such as nuclear delocalization, anharmonic zero-point energy, and tunneling into energy calculations, geometry optimizations, and dynamics. NEO coupled cluster (NEO-CC) methods recover correlation to an extent that enables prediction of accurate ground-state properties for moderately sized systems. Herein, NEO-CCSD­(T), which includes all relevant single and double electron and nucleus excitations, as well as perturbative triple excitations, is formulated with the nuclear Hartree product representation for an arbitrary number of quantum nuclei. Compared to the nuclear Slater determinant representation, iterative convergence of the *t*-amplitude equations, as well as the preceding NEO Hartree–Fock procedure, is significantly faster for multiple quantum protons. Moreover, perturbative corrections in methods such as NEO second-order Møller–Plesset perturbation theory and NEO-CCSD­(T) are more accurate using the nuclear Hartree product representation, most likely due to more physically reasonable virtual orbitals. Electronic frozen natural orbital truncation is implemented to extend the accessible system size while maintaining accuracy. These combined methods are used to calculate interaction energies of systems with multiple hydrogen bonds, as well as energies of water clusters with up to 24 quantum protons. In addition, NEO-CCSD­(T) with the nuclear Hartree product representation is used to predict hydrogen/deuterium geometric isotope effects, incorporating the necessary anharmonic zero-point energy, for FHF^–^, a water dimer, and HF clusters of up to five molecules. These examples show that NEO-CCSD­(T) with the nuclear Hartree product representation is an efficient and accurate method for incorporating nuclear quantum effects into ground-state calculations.

## Graphical abstract

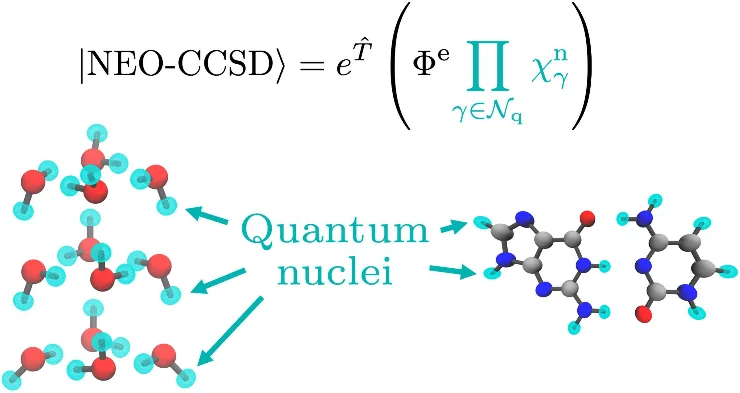

## 1 Introduction

The nuclear–electronic orbital (NEO) framework^1–3^ allows for the incorporation of nuclear quantum effects directly into quantum chemical calculations. In the NEO framework, select nuclei, typically protons, are treated quantum mechanically on the same level as electrons. This approach includes properties such as nuclear delocalization, anharmonic zero-point energy (ZPE), nuclear tunneling, and non-Born–Oppenheimer effects between the electrons and quantum nuclei directly in single-point energy calculations, geometry optimizations, vibrational or vibronic frequency calculations,^4–7^ reaction paths,^8^ and dynamics.^5,9–11^ For these purposes, a variety of wave function NEO methods, as well as NEO density functional theory (NEO-DFT) methods, have been developed. These NEO methods have been used to incorporate nuclear quantum effects at a relatively low cost for calculations on organic molecules and catalysts,^12,13^ biological systems,^14^ and materials.^15^ We also point out that NEO methods can be viewed as part of the multicomponent quantum chemistry field for treating multiple types of particles quantum mechanically.^2,16–25^

NEO Hartree–Fock (NEO-HF) is the most fundamental NEO method.^1^ However, analogous to conventional electronic structure theory, in NEO-HF the quantum mechanically treated particles feel only the mean field of the other quantum particles. Electron–electron correlation is known to be important for attaining sufficient accuracy in quantum chemistry. The lack of electron–proton correlation leads to highly over-localized proton densities and therefore unreliable molecular properties.^2^ One pathway for recovering the missing correlation is through NEO-DFT^26^ using electron–proton correlation functionals^27–29^ that have been developed to remedy these issues. For hydrogen tunneling problems, static correlation is critical and can be included with NEO multistate DFT (NEO-MSDFT).^10,30,31^ Excited states and vibronic frequencies can be calculated through NEO time-dependent DFT (NEO-TDDFT).^4,32^ However, NEO-DFT is not systematically improvable and is not able to reliably predict properties that are dependent on quantitatively accurate proton densities.^3^

NEO wave function methods are systematically improvable and are typically parameter free. A variety of these methods have been developed for recovering dynamic correlation energy for the ground state with different balances of cost versus accuracy. Such methods include NEO second-order Møller–Plesset perturbation theory^33,34^ (NEO-MP2) and its orbital optimized^35,36^ (NEO-OOMP2) counterpart and NEO coupled cluster (NEO-CC) methods using full excitations^33,37–39^ as well as perturbative corrections.^38–40^ For handling problems with important static correlation such as hydrogen tunneling, NEO multiconfigurational self-consistent field^1^ (NEO-MCSCF) and NEO multireference configuration interaction^41,42^ (NEO-MRCI) have been developed. Excited vibronic states have also been studied through NEO second-order coupled cluster (NEO-CC2),^43^ NEO equation of motion coupled cluster (NEO-EOM-CC),^44–46^ and NEO-MRCI.^41,42^

For systems without significant static correlation, NEO coupled cluster (NEO-CC) methods are favored for recovering dynamic correlation and predicting accurate ground state properties due to the size-consistency and implicit inclusion of higher-order excitations in comparison with CI methods truncated at the same excitation level.^47^ Previously, NEO-CC with singles and doubles (NEO-CCSD) was developed and used to calculate relative energies of protonated water cluster isomers, proton densities, proton affinities, and vibrationally averaged geometries.^37,48–50^ Because electron–proton correlation is especially important within the NEO framework,^1–3^ NEO-CCSD with electron–electron–proton triple excitations (NEO-CCSDT_eep_) was developed and was found to predict more accurate proton affinities than NEO-CCSD.^38,39^ However, due to the high expense of this method, perturbative triples were developed as an alternative within the NEO-CCSD­(T) method and were found to predict proton affinities with nearly the same accuracy as NEO-CCSDT_eep_.^39^ This work also showed that the NEO-CCSD­(T) method reproduces experimental proton affinities within chemical accuracy at the extrapolated complete basis set limit.^39^ Additionally, NEO-CCSD­(T) was used to calculate relative energies of protonated water tetramer isomers, with all nine protons treated quantum mechanically, and a decomposition of the correlation energy indicated that missing higher excitations^51,52^ were unlikely to contribute significantly to the correlation energy in most cases.^39^ These results illustrate the promise of NEO-CCSD­(T) as the “gold standard” NEO method for recovering dynamic correlation for ground state systems, analogous to the role of CCSD­(T)^53^ in conventional electronic structure theory.

Typically, NEO methods have comparable computational cost as their conventional electronic counterparts. Because most systems are composed of many more electrons than quantum nuclei and require more electronic basis functions than nuclear basis functions, the number of electrons and electronic basis functions usually determines the cost. However, many methods, such as HF and CC, require the solution of iterative equations. The introduction of additional degrees of freedom related to the quantum nuclei into these methods can cause much slower iterative convergence. Recently, substantial improvements to the iterative convergence of the NEO self-consistent field (SCF) procedure have been achieved.^54^ One of these improvements is based on the nuclear Hartree product representation within the NEO wavefunction.^54,55^ The proton–proton exchange terms are typically eight to ten orders of magnitude smaller than their electronic counterparts^2^ and are usually smaller than the SCF convergence threshold. Thus, neglecting proton–proton exchange terms has no impact on the accuracy of the energy. Moreover, the nuclear Hartree product representation has been shown to decrease the number of SCF iterations by a factor of four to six for the systems studied^54^ and thus leads to much faster convergence.

Herein, we formulate NEO-CC using the nuclear Hartree product (HP) representation within the reference wavefunction for an arbitrary number of distinguishable quantum nuclei. This formalism is applicable to both bosonic and fermionic nuclei, as long as the exchange interactions between fermions are negligible, as found for protons, and assuming single occupation of each nuclear orbital. Previously, NEO-CC using the nuclear HP representation had only been implemented for the specific case of two quantum hydrogen nuclei.^56^ After submission of this paper, an example of NEO-CC calculations on a system with three distinguishable quantum protons was published.^57^ Our general formulation, combined with an efficient implementation, provides the flexibility to study systems with many quantum nuclei, easily treating systems with more than 20 quantum nuclei, thereby greatly expanding the range of potential applications.

Furthermore, we perform analyses aimed at understanding the improvements arising from combining NEO-CC methods with the nuclear HP representation. Our results show that using the nuclear HP representation significantly speeds up iterative convergence for the *t*-amplitude equations in NEO-CCSD. We also show that the NEO-CCSD­(T) perturbative triples correction and NEO-MP2 correction^34^ are more accurate with the nuclear HP representation, most likely because of more physically meaningful virtual orbitals. Our analysis provides insight into the underlying physical basis for the substantial speed-up and improvement of the perturbative corrections. In order to handle larger systems with larger electronic basis sets, we explore the use of frozen natural orbital (FNO)^58,59^ truncation. After careful benchmarking, these enhanced NEO-CCSD­(T) methods are used to calculate interaction energies and geometric isotope effects for systems with multiple hydrogen bonds, as well as energies of water clusters with up to 24 quantum nuclei. In this work, only hydrogen and deuterium nuclei are treated quantum mechanically. However, the formulation presented here is applicable to any set of quantum nuclei, each with any charge and mass. These improvements to NEO-CCSD­(T) allow for even higher accuracy and efficiency for what can be viewed as the current “gold standard” method for ground-state systems within the field of multicomponent quantum chemistry.

## 2 Theory

Within the NEO-HF ansatz, the wave function can be expressed as

1 $$\Psi^{\mathrm{SD}}\left(x^{e},x^{n}\right)=\Phi^{e}\left(x^{e}\right)\Phi^{n}\left(x^{n}\right)$$

where Φ^e^(**x** ^e^) and Φ^n^(**x** ^n^) are electronic and nuclear Slater determinants (SDs) of spin orbitals, respectively. Herein, **x** ^e^ and **x** ^n^ are the collective spatial and spin coordinates of the electrons and quantum nuclei, respectively. The Slater determinant representation has typically been used in the case of quantum protons, as they are formally indistinguishable fermions. However, as nuclear orbitals are highly localized in molecular systems, exchange interactions between quantum protons are very small and can be neglected by treating all nuclei as distinguishable particles. This approximation leads to the following nuclear Hartree product (HP) representation^54,55^ of the NEO-HF wave function:

2 $$\Psi^{\mathrm{HP}}\left(x^{e},x^{n}\right)=\Phi^{e}\left(x^{e}\right)\underset{\gamma \in N_{q}}{\prod }\chi_{\gamma ,1}^{n}\left(x_{\gamma }^{n}\right)$$

Here, $N_{q}$ is the set of nuclei treated quantum mechanically, and $\chi_{\gamma ,P}^{n}\left(x_{\gamma }^{n}\right)$ is the nuclear spin orbital for nucleus γ and orbital label *P*. Here *P* = 1 corresponds to the lowest-energy orbital for that nucleus, and *P* > 1 corresponds to the higher-energy orbitals.

This representation leads to the following Hartree–Fock equations for the orbital energy $\varepsilon_{i}^{e}$ of electronic spin orbital $\chi_{i}^{e}$ and the orbital energy $\varepsilon_{\gamma ,1}^{n}$ of the nuclear spin orbital $\chi_{\gamma ,1}^{n}$ :

3a $$\left[h^{e}\left(x^{e}\right)+\underset{\begin{array}{c} j\in S^{e\left(o\right)}\\ j\neq i \end{array}}{\sum }\left[J_{j}^{\mathrm{ee}}\left(x^{e}\right)-K_{j}^{e}\left(x^{e}\right)\right]+\underset{\eta \in N_{q}}{\sum }J_{\eta }^{\mathrm{en}}\left(x^{e}\right)\right]\chi_{i}^{e}\left(x^{e}\right)=\varepsilon_{i}^{e}\chi_{i}^{e}\left(x^{e}\right)$$

3b $$\left[h_{\gamma }^{n}\left(x_{\gamma }^{n}\right)+\underset{\begin{array}{c} \eta \in N_{q}\\ \eta \neq \gamma \end{array}}{\sum }J_{\gamma ,\eta }^{\mathrm{nn}}\left(x_{\gamma }^{n}\right)+\underset{j\in S^{e\left(o\right)}}{\sum }J_{\gamma ,j}^{\mathrm{ne}}\left(x_{\gamma }^{n}\right)\right]\chi_{\gamma ,1}^{n}\left(x_{\gamma }^{n}\right)=\varepsilon_{\gamma ,1}^{n}\;\chi_{\gamma ,1}^{n}\left(x_{\gamma }^{n}\right)$$

Here, *h* ^e^ is the electronic core Hamiltonian operator, $J_{j}^{\mathrm{ee}}$ is the electronic Coulomb operator associated with an electron in spin orbital $\chi_{j}^{e}$ , and $K_{j}^{e}$ is the electronic exchange operator associated with spin orbital $\chi_{j}^{e}$ , as in conventional electronic structure. In addition, $h_{\gamma }^{n}$ is the core Hamiltonian operator for nucleus *γ*, $J_{\eta }^{\mathrm{en}}$ is the Coulomb operator associated with a nucleus *η* in spin orbital $\chi_{\eta ,1}^{n}$ and the charge product −*Z*_*η*_, $J_{\gamma ,\eta }^{\mathrm{nn}}$ is the Coulomb operator associated with a nucleus *η* in $\chi_{\eta ,1}^{n}$ and the charge product *Z*_*γ*_ *Z*_*η*_, and $J_{\gamma ,j}^{\mathrm{ne}}$ is the Coulomb operator associated with an electron in spin orbital $\chi_{j}^{e}$ and the charge product −*Z*_*γ*_. Here, *Z*_*γ*_ is the charge of nucleus *γ*, and the charge product refers to the product of the charges of a nucleus and an electron or two nuclei. For notational simplicity, charge is incorporated into operators such that there are no additional prefactors. Moreover, $S^{e\left(o\right)}$ is the set of all occupied electronic orbital indices, and eq 3a is composed of *N* ^e^ equations, where *N* ^e^ is the number of electrons and $i\in S^{e\left(o\right)}$ .

From eqs 3a and 3b, we can straightforwardly define an electronic Fock operator *F* ^e^, a nuclear Fock operator $F_{\gamma }^{n}$ , and corresponding eigenvalue equations as

4a $$F^{e}\left(x^{e}\right)=h^{e}\left(x^{e}\right)+\underset{j\in S^{e\left(o\right)}}{\sum }\left[J_{j}^{\mathrm{ee}}\left(x^{e}\right)-K_{j}^{e}\left(x^{e}\right)\right]+\underset{\eta \in N_{q}}{\sum }J_{\eta }^{\mathrm{en}}\left(x^{e}\right)$$

4b $$F_{\gamma }^{n}\left(x_{\gamma }^{n}\right)=h_{\gamma }^{n}\left(x_{\gamma }^{n}\right)+\underset{\begin{array}{c} \eta \in N_{q}\\ \eta \neq \gamma \end{array}}{\sum }J_{\gamma ,\eta }^{\mathrm{nn}}\left(x_{\gamma }^{n}\right)+\underset{j\in S^{e\left(o\right)}}{\sum }J_{\gamma ,j}^{\mathrm{ne}}\left(x_{\gamma }^{n}\right)$$

4c $$F^{e}\left(x^{e}\right)\chi_{p}^{e}\left(x^{e}\right)=\varepsilon_{p}^{e}\chi_{p}^{e}\left(x^{e}\right)$$

4d $$F_{\gamma }^{n}\left(x_{\gamma }^{n}\right)\chi_{\gamma ,P}^{n}\left(x_{\gamma }^{n}\right)=\varepsilon_{\gamma ,P}^{n}\chi_{\gamma ,P}^{n}\left(x_{\gamma }^{n}\right)$$

Defining the set of all orbital indices for the electrons as $S^{e}$ and the set of all orbital indices for nucleus *γ* as $S_{\gamma }^{n}$ , $p\in S^{e}$ , and $P\in S_{\gamma }^{n}$ . In the NEO-HF nuclear HP representation, the ground state corresponds to *P* = 1 for all nuclei. Equation 4d is composed of *N* ^n^ equations, where *N* ^n^ is the number of quantum nuclei. NEO-HF using the nuclear HP representation with the nuclear Fock operator defined above was found to converge more quickly than NEO-HF using the nuclear SD representation for multiple quantum protons.^54^ When self-Coulomb and self-exchange terms are included in the nuclear Fock operator within the nuclear HP representation, the NEO-HF energy is unchanged, but convergence is more similar to the convergence for the nuclear SD representation.^54^ Inclusion of the self-Coulomb and self-exchange terms, which are also implicitly included in the nuclear SD representation, alters the virtual orbitals^34^ and leads to slow convergence. See the Supporting Information for more information about the impact of the self-Coulomb and self-exchange terms.

Next, we will formulate NEO-CC within the nuclear HP representation for the Fock operator $F_{\gamma }^{n}$ defined in eq 4b to obtain improved convergence of the *t*-amplitude equations compared to the convergence for the nuclear SD representation. This formulation will also offer a more accurate perturbative triples correction. *F* ^e^ and $F_{\gamma }^{n}$ can be written in second quantization as

5a $${\mathrm{F̂}}^{\mathrm{e,}\left(\mathrm{unordered}\right)}=\underset{pq\in S^{e}}{\sum }{\left(F^{e}\right)}_{q}^{p}a_{p}^{q}=\underset{pq\in S^{e}}{\sum }\left({\left(h^{e}\right)}_{q}^{p}+\underset{j\in S^{e\left(o\right)}}{\sum }{\left({\mathrm{g̅}}^{\mathrm{ee}}\right)}_{qj}^{pj}+\underset{\eta \in N_{q}}{\sum }\underset{J\in S_{\eta }^{n\left(o\right)}}{\sum }{\left(g_{\eta }^{\mathrm{en}}\right)}_{qJ}^{pJ}\right)a_{p}^{q}$$

5b $${\mathrm{F̂}}_{\gamma }^{\mathrm{n,}\left(\mathrm{unordered}\right)}=\underset{PQ\in S_{\gamma }^{n}}{\sum }{\left(F_{\gamma }^{n}\right)}_{Q}^{P}a_{P}^{Q}=\underset{PQ\in S_{\gamma }^{n}}{\sum }\left({\left(h_{\gamma }^{n}\right)}_{Q}^{P}+\underset{\begin{array}{c} \eta \in N_{q}\\ \eta \neq \gamma \end{array}}{\sum }\underset{J\in S_{\eta }^{n\left(o\right)}}{\sum }{\left(g_{\gamma \eta }^{\mathrm{nn}}\right)}_{QJ}^{PJ}+\underset{j\in S^{e\left(o\right)}}{\sum }{\left(g_{\gamma }^{\mathrm{ne}}\right)}_{Qj}^{Pj}\right)a_{P}^{Q}$$

Occupied and virtual subsets of the nuclear orbital indices are $S_{\gamma }^{n\left(o\right)}$ and $S_{\gamma }^{n\left(v\right)}$ , respectively, and electronic occupied and virtual subsets are defined analogously. Note that since $S_{\eta }^{n\left(o\right)}$ contains only a single index for each nucleus *η*, the summations $\underset{J\in S_{\eta }^{n\left(o\right)}}{\sum }$ are over only a single occupied orbital index. Here, $a_{p_{1}p_{2}...p_{n}}^{q_{1}q_{2}...q_{n}}=a_{q_{1}}^{†}a_{q_{2}}^{†}...a_{q_{n}}^{†}a_{p_{n}}...a_{p_{2}}a_{p_{1}}$ are excitation operators expressed as strings of creation and annihilation operators. Moreover, ${\left(h^{e}\right)}_{q}^{p}=\left\langle q\right|h^{e}\left|p\right\rangle$ , ${\left(h_{\gamma }^{n}\right)}_{Q}^{P}=\left\langle Q\right|h_{\gamma }^{n}\left|P\right\rangle$ , ${\left({\mathrm{g̅}}^{\mathrm{ee}}\right)}_{rs}^{pq}=\left\langle rs\left|r_{12}^{-1}\right|pq\right\rangle -\left\langle rs\left|r_{12}^{-1}\right|qp\right\rangle$ , ${\left(g_{\gamma \eta }^{\mathrm{nn}}\right)}_{QS}^{PR}=\left\langle QS\left|Z_{\gamma }Z_{\eta }r_{12}^{-1}\right|PR\right\rangle$ , and ${\left(g_{\gamma }^{\mathrm{en}}\right)}_{qQ}^{pP}={\left(g_{\gamma }^{\mathrm{ne}}\right)}_{Qq}^{Pp}=-\left\langle qQ\left|Z_{\gamma }r_{12}^{-1}\right|pP\right\rangle$ , where *Z*_γ_ is the charge of nucleus γ, and the two-particle integrals are defined using standard physicist’s notation.

The NEO Hamiltonian written in second quantization and normal-ordered with respect to the nuclear HP NEO-HF reference state, |0^e^0^n^⟩ = Ψ^HP^(**x** ^e^, **x** ^n^), is

6 ĤNEO=∑pq∈Se(Fe)qpãpq+14∑pqrs∈Se(g̅ee)rspqãpqrs+∑γ∈Nq∑PQ∈Sγn(Fγn)QPãPQ+∑γ,η∈Nqγ<η∑PQ∈Sγn∑RS∈Sηn(gγηnn)QSPRãPRQS+∑γ∈Nq∑pq∈Se∑PQ∈Sγn(gγen)qQpPãpPqQ

where operators with tildes are normal-ordered with respect to |0^e^0^n^⟩. For simplicity, this form of the NEO Hamiltonian can be written as

7 $${Ĥ}_{\mathrm{NEO}}={\mathrm{F̂}}^{e}+{ĝ}^{\mathrm{ee}}+\underset{\gamma \in N_{q}}{\sum }{\mathrm{F̂}}_{\gamma }^{n}+\underset{\begin{array}{c} \gamma ,\eta \in N_{q}\\ \gamma <\eta \end{array}}{\sum }{ĝ}_{\gamma \eta }^{\mathrm{nn}}+\underset{\gamma \in N_{q}}{\sum }{ĝ}_{\gamma }^{\mathrm{en}}$$

The NEO-CCSD wave function can be written as

8 $$\left|\mathrm{NEO‐CCSD}\right\rangle =e^{{\mathrm{T̂}}_{\mathrm{NEO‐CCSD}}}\left|0^{e}0^{n}\right\rangle$$

where ${\mathrm{T̂}}_{\mathrm{NEO‐CCSD}}$ is the truncated multicomponent cluster operator producing electronic and nuclear excitations. If we neglect double nuclear excitations, which have been found to contribute negligibly to the correlation energy for molecular systems in the case of quantum protons,^39^ this operator can be written as

9 $${\mathrm{T̂}}_{\mathrm{NEO‐CCSD}}=\underset{i\in S^{e\left(o\right)}}{\sum }\underset{a\in S^{e\left(v\right)}}{\sum }{\left(t^{e}\right)}_{a}^{i}{ã}_{i}^{a}+\frac{1}{4}\underset{ij\in S^{e\left(o\right)}}{\sum }\underset{ab\in S^{e\left(v\right)}}{\sum }{\left(t^{\mathrm{ee}}\right)}_{ab}^{ij}{ã}_{ij}^{ab}+\underset{\gamma \in N_{q}}{\sum }\underset{I\in S_{\gamma }^{n\left(o\right)}}{\sum }\underset{A\in S_{\gamma }^{n\left(v\right)}}{\sum }{\left(t_{\gamma }^{n}\right)}_{A}^{I}{ã}_{I}^{A}+\underset{\gamma \in N_{q}}{\sum }\underset{i\in S^{e\left(o\right)}}{\sum }\underset{a\in S^{e\left(v\right)}}{\sum }\underset{I\in S_{\gamma }^{n\left(o\right)}}{\sum }\underset{A\in S_{\gamma }^{n\left(v\right)}}{\sum }{\left(t_{\gamma }^{\mathrm{en}}\right)}_{aA}^{iI}{ã}_{iI}^{aA}$$

Here, the various *t* parameters are the *t*-amplitudes, which can be obtained by solving the relevant equations. As mentioned above, $S_{\gamma }^{n\left(o\right)}$ contains only a single index for each nucleus *γ* and, therefore, the summations $\underset{I\in S_{\gamma }^{n\left(o\right)}}{\sum }$ are over only a single index. For simplicity, this operator can be rewritten as

10 $${\mathrm{T̂}}_{\mathrm{NEO‐CCSD}}={\mathrm{T̂}}^{e}+{\mathrm{T̂}}^{\mathrm{ee}}+\underset{\gamma \in N_{q}}{\sum }{\mathrm{T̂}}_{\gamma }^{n}+\underset{\gamma \in N_{q}}{\sum }{\mathrm{T̂}}_{\gamma }^{\mathrm{en}}$$

The correlation energy and *t*-amplitudes are found through the following equations:

11a $$E_{\mathrm{NEO}}=\left\langle 0^{e}0^{n}\left|{\mathrm{H̅}}_{\mathrm{NEO}}\right|0^{e}0^{n}\right\rangle$$

11b $$0=\left\langle \mu^{e}\left|{\mathrm{H̅}}_{\mathrm{NEO}}\right|0^{e}0^{n}\right\rangle$$

11c $$0=\left\langle \mu^{\mathrm{ee}}\left|{\mathrm{H̅}}_{\mathrm{NEO}}\right|0^{e}0^{n}\right\rangle$$

11d $$0=\left\langle \mu_{\kappa }^{n}\left|{\mathrm{H̅}}_{\mathrm{NEO}}\right|0^{e}0^{n}\right\rangle$$

11e $$0=\left\langle \mu_{\kappa }^{\mathrm{en}}\left|{\mathrm{H̅}}_{\mathrm{NEO}}\right|0^{e}0^{n}\right\rangle$$

where $\left\langle \mu^{e}\right|=\left\langle 0^{e}0^{n}\right|a_{a}^{i}$ , $\left\langle \mu^{\mathrm{ee}}\right|=\left\langle 0^{e}0^{n}\right|a_{ab}^{ij}$ , $\left\langle \mu_{\kappa }^{n}\right|=\left\langle 0^{e}0^{n}\right|a_{A}^{I}$ , and $\left\langle \mu_{\kappa }^{\mathrm{en}}\right|=\left\langle 0^{e}0^{n}\right|a_{aA}^{iI}$ for $i\in S^{e\left(o\right)}$ , $a\in S^{e\left(v\right)}$ , $I\in S_{\kappa }^{n\left(o\right)}$ , and $A\in S_{\kappa }^{n\left(v\right)}$ . Moreover, *E*_NEO_ is the correlation energy, and ${\mathrm{H̅}}_{\mathrm{NEO}}=e^{-{\mathrm{T̂}}_{\mathrm{NEO‐CCSD}}}{Ĥ}_{\mathrm{NEO}}e^{{\mathrm{T̂}}_{\mathrm{NEO‐CCSD}}}$ . Equations 11d and 11e must be solved for each quantum nucleus *κ*. Obtaining programmable equations for these equations using Wick’s theorem^60^ is not straightforward because an arbitrary number of nuclei leads to an arbitrary number of unique index spaces.

Here, a simple procedure was used to obtain these programmable equations. This procedure could also be used to obtain programmable equations when double nuclear excitations or higher excitation levels are included. The only nuclei appearing in a given term after usage of Wick’s theorem are those from the associated term in the Hamiltonian or from the bra in eqs 11a–11e. Thus, if we rewrite the Hamiltonian with respect to the nuclei from the bra, if there are any, and then select the electronic terms and one term from each summation over nuclei or pairs of nuclei, we obtain a representative Hamiltonian that can be used to obtain programmable equations using Wick’s theorem. The associated representative cluster operator must contain any distinct terms within the excitation level that can connect to the representative Hamiltonian. This representative cluster operator does not result in double counting because the nuclei present in a term are strictly determined from the nuclei in the bra and the associated term of the Hamiltonian. Then, after using Wick’s theorem, the summations over nuclei or pairs of nuclei, which were previously removed, must be added back to the programmable equations.

First, we go through this procedure for eqs 11a–11c. These equations do not contain a bra with a nuclear excitation, and therefore the Hamiltonian does not need to be rewritten. For these equations, the representative Hamiltonian ${Ĥ}_{\mathrm{NEO}}^{\prime }$ , which selects the electronic terms and one term from each summation shown in eq 7, and the associated representative cluster operator, ${\mathrm{T̂}}_{\mathrm{NEO‐CCSD}}^{\prime }$ , both involve only two nuclei, *γ* and *η*, and can be written as

12a $${Ĥ}_{\mathrm{NEO}}^{\prime }={\mathrm{F̂}}^{e}+{ĝ}^{\mathrm{ee}}+{\mathrm{F̂}}_{\gamma }^{n}+{ĝ}_{\gamma \eta }^{\mathrm{nn}}+{ĝ}_{\gamma }^{\mathrm{en}}$$

12b $${\mathrm{T̂}}_{\mathrm{NEO‐CCSD}}^{\prime }={\mathrm{T̂}}^{e}+{\mathrm{T̂}}^{\mathrm{ee}}+{\mathrm{T̂}}_{\gamma }^{n}+{\mathrm{T̂}}_{\eta }^{n}+{\mathrm{T̂}}_{\gamma }^{\mathrm{en}}+{\mathrm{T̂}}_{\eta }^{\mathrm{en}}$$

Wick’s theorem can be easily applied to eqs 11a–11c using eqs 12a and 12b to obtain programmable equations because there are only two occupied and two virtual nuclear spaces that need to be considered. Then, after using Wick’s theorem, for any term that involves only the *γ* nucleus, a summation $\underset{\gamma \in N_{q}}{\sum }$ is used. For any term that involves both the *γ* and *η* nuclei, a summation $\underset{\begin{array}{l} \gamma ,\eta \in N_{q},\gamma <\eta \end{array}}{\sum }$ is used.

Next, we go through this procedure for eqs 11d and 11e. Since these equations contain a bra with a single nuclear excitation for nucleus *κ*, we rewrite the Hamiltonian in relation to this nucleus *κ* explicitly using the set of remaining nuclei, $N_{r}=N_{q}\backslash \left\{\kappa \right\}$ , as follows:

13 $${Ĥ}_{\mathrm{NEO}}={\mathrm{F̂}}^{e}+{ĝ}^{\mathrm{ee}}+{\mathrm{F̂}}_{\kappa }^{n}+\underset{\xi \in N_{r}}{\sum }{\mathrm{F̂}}_{\xi }^{n}+\underset{\xi \in N_{r}}{\sum }{ĝ}_{\kappa \xi }^{\mathrm{nn}}+\underset{\begin{array}{c} \xi ,\eta \in N_{r}\\ \xi <\eta \end{array}}{\sum }{ĝ}_{\xi \eta }^{\mathrm{nn}}+{ĝ}_{\kappa }^{\mathrm{en}}+\underset{\xi \in N_{r}}{\sum }{ĝ}_{\xi }^{\mathrm{en}}$$

Note that the summation $\underset{\begin{array}{l} \xi ,\eta \in N_{r},\xi <\eta \end{array}}{\sum }{ĝ}_{\xi \eta }^{\mathrm{nn}}$ cannot be involved because this summation only involves $N_{r}$ nuclei, and the truncated cluster operator contains no term that can connect an $N_{r}$ nucleus to the *κ* nucleus to produce a *κ* nucleus excitation (as double nuclear excitations were excluded). Thus, for these equations, the representative Hamiltonian ${Ĥ}_{\mathrm{NEO}}^{\prime \prime }$ , which selects the electronic terms and one term from each summation shown in eq 13 (except $\underset{\begin{array}{l} \xi ,\eta \in N_{r},\xi <\eta \end{array}}{\sum }{ĝ}_{\xi \eta }^{\mathrm{nn}}$ ), and the associated representative cluster operator, ${\mathrm{T̂}}_{\mathrm{NEO‐CCSD}}^{\prime \prime }$ , both involve only two nuclei, *κ* and *ξ*, and can be written as

14a $${Ĥ}_{\mathrm{NEO}}^{\prime \prime }={\mathrm{F̂}}^{e}+{ĝ}^{\mathrm{ee}}+{\mathrm{F̂}}_{\kappa }^{n}+{\mathrm{F̂}}_{\xi }^{n}+{ĝ}_{\kappa \xi }^{\mathrm{nn}}+{ĝ}_{\kappa }^{\mathrm{en}}+{ĝ}_{\xi }^{\mathrm{en}}$$

14b $${\mathrm{T̂}}_{\mathrm{NEO‐CCSD}}^{\prime \prime }={\mathrm{T̂}}^{e}+{\mathrm{T̂}}^{\mathrm{ee}}+{\mathrm{T̂}}_{\kappa }^{n}+{\mathrm{T̂}}_{\xi }^{n}+{\mathrm{T̂}}_{\kappa }^{\mathrm{en}}+{\mathrm{T̂}}_{\xi }^{\mathrm{en}}$$

Again, Wick’s theorem can be easily applied to eqs 11d and 11e using eqs 14a and 14b to obtain programmable equations because only two occupied and two virtual nuclear spaces need to be considered. Then, in the programmable equations for any term involving the ξ nucleus, a summation $\underset{\xi \in N_{r}}{\sum }$ is used. Here, a modified version of existing open source code was used to apply Wick’s theorem algebraically.^61^ The derived programmable spin orbital equations are provided in the Supporting Information. This strategy can also be used to obtain programmable equations for an arbitrary number of nuclei while including other excitations, such as double nuclear excitations or higher excitations. As an example, we have used this strategy to set up the generation of the energy and *t*-amplitude programmable equations when double nuclear excitations are included (see pages S13–S15 in the Supporting Information).

## 3 Computational Details

All NEO calculations performed herein treat the electrons and all hydrogen nuclei quantum mechanically, unless otherwise noted. We implemented the NEO-CC methods described above in a development version of the Q-Chem package.^62^ The equations are spin-adapted to treat electrons as restricted closed-shell, and nuclei are treated as high spin when using the nuclear SD representation. The frozen core approximation was used for the electrons. Density fitting was used to approximate the four-center molecular orbital basis integrals to reduce memory usage.^49^ Our calculations used the aug-cc-pVXZ electronic basis sets^63,64^ and corresponding rimp2-aug-cc-pVXZ auxiliary electronic basis sets,^65–67^ where X is T, Q, or 5. For protons, our NEO calculations used the PB4-F2 protonic basis set^68^ and even-tempered 8s8p8d8f auxiliary protonic basis set^32^ with exponents ranging from 2 $\sqrt{2}$ to 32. For deuterons, the exponents of the protonic basis set and auxiliary protonic basis set were scaled by $\sqrt{2}$ to account for the mass ratio. When NEO calculations were performed at geometries obtained using conventional electronic methods, the nuclear basis function centers were placed at the optimized position of the classical nucleus. When geometries were optimized using NEO methods, the nuclear basis function center positions were also optimized.

The initial guess for the NEO-CCSD *t*-amplitudes was obtained from a NEO-MP2 calculation. A quasi-Newton update of the form $t_{\left(n+1\right)}^{\mu }=t_{\left(n\right)}^{\mu }+\Delta t_{\left(n\right)}^{\mu }$ was used, where $t_{\left(n\right)}^{\mu }$ is a *t*-amplitude at iteration *n* for excitation *μ* and $\Delta t_{\left(n\right)}^{\mu }$ is the update to that *t*-amplitude. The update was calculated as $\Delta t_{\left(n\right)}^{\mu }={\left(-\varepsilon^{\mu }\right)}^{-1}\Omega_{\left(n\right)}^{\mu }$ , where $\Omega_{\left(n\right)}^{\mu }$ is the corresponding residual, i.e., the left-hand side of eqs 11b–11e for $t_{\left(n\right)}^{\mu }$ , and ε^*μ*^ is the sum of orbital energy differences for the excitation.^69^ The *t*-amplitudes were updated sequentially by excitation type in the order *t* ^e^, *t* ^n^, *t* ^ee^, and *t* ^en^. Direct inversion of the iterative subspace (DIIS) was used to accelerate the convergence of the *t*-amplitude equations. The DIIS algorithm used a combined *t*-amplitude vector containing all $t_{\left(n\right)}^{\mu }$ and used all $\Delta t_{\left(n\right)}^{\mu }$ to construct the error vector.

To benchmark the NEO-CCSD­(T) method, we computed the energies of FHF^–^ and FDF^–^ using the Fourier Grid Hamiltonian (FGH) method^70,71^ at the CCSD­(T) level. These reference energies, denoted the FGH/CCSD­(T) energies, were computed for a series of F–F distances. For each F–F distance, an FGH calculation was performed to compute the ZPE associated with H or D. For the FGH calculations, single-point energies were calculated at the CCSD­(T)/aug-cc-pVTZ level of theory as the hydrogen nucleus was moved on a three-dimensional grid with the F atoms fixed. The 32 × 32 × 32 point grid corresponding to a 1 × 1 × 1 Å box was centered at the midpoint between the F atoms. The electronic basis function center was moved along with the hydrogen nucleus point charge. The vibrational wave functions and energy levels of the H or D nucleus were obtained by solving the time-independent Schrödinger equation for this three-dimensional potential energy surface. The FGH/CCSD­(T) reference energy was then computed for this F–F distance by adding the resulting ZPE to the electronic energy computed at the CCSD­(T)/aug-cc-pV5Z level of theory with the hydrogen at the midpoint between the two F atoms.

## 4 Results

### 4.1 Nuclear Hartree Product Representation Speeds Up Iterative Convergence of NEO-CCSD

Table 1 shows the number of iterations required to converge the *t*-amplitude equations for NEO-CCSD calculations performed on a set of small molecular systems with multiple quantum protons for the nuclear HP representation and the nuclear SD representation. For a fair comparison, the nuclear SD calculations also excluded double proton excitations. These calculations used the aug-cc-pVTZ electronic basis set. The geometries were optimized at the conventional CCSD/aug-cc-pVTZ level of theory, except for the protonated water tetramers, which were optimized at the CCSD­(T)/aug-cc-pVTZ level of theory obtained from ref 72. As typically the DIIS maximum history is truncated to reduce memory usage, a maximum DIIS history of seven was used. Results with an untruncated DIIS history are provided in the Supporting Information. For nuclear SD calculations using a truncated DIIS history, performing a DIIS extrapolation only after every three iterations offered greatly improved convergence compared to extrapolating after every iteration. Thus, Table 1 shows the improved results obtained by performing a DIIS extrapolation only after every three iterations for the nuclear SD calculations. For the nuclear HP calculations and the nuclear SD calculations in Table S1 in the Supporting Information, a DIIS extrapolation was performed after every iteration. For a fair comparison, calculations were considered to be converged when both the energy and the RMSE of the *t*-amplitudes were within 10^–6^ of a very tightly converged calculation performed prior to this analysis. This convergence criterion was only used for the analysis of the number of iterations.

**Table 1:** Number of Iterations to Converge the NEO-CCSD *t*-Amplitude Equations

system	SD	HP	ratio
H^+^(H_2_O)_4_ (Eigen)	72	10	7.2
H^+^(H_2_O)_4_ (ring)	66	10	6.6
H^+^(H_2_O)_4_ (*cis*-Zundel)	76	13	5.8
H^+^(H_2_O)_4_ (*trans*-Zundel)	78	13	6.0
HCCH	66	10	6.6
H_2_O	72	11	6.5
CH_3_OH	66	13	5.1
CH_3_NH_2_	66	13	5.1
HCOOH	66	13	5.1
CH_2_O	69	11	6.3
(H_2_O)_2_	69	11	6.3
H^+^(H_2_O)_2_	69	12	5.8
mean	70	12	6.0

The nuclear HP representation offers a large and consistent speedup in iterative convergence of about six times over the nuclear SD representation. A similar speedup occurs even when the full DIIS history is kept (Table S1), and this speedup does not depend strongly on the convergence threshold (Table S2). The number of iterations required for convergence for the case of nuclear HP calculations is within the typical range expected for conventional electronic CCSD calculations, whereas the nuclear SD calculations require more iterations. The calculations using the nuclear HP representation also benefit from the dramatic speedup in convergence of the NEO-HF equations.^54^ Thus, NEO-CC using the nuclear Hartree product representation as formulated here provides a notable computational efficiency improvement for NEO-CC calculations with multiple quantum nuclei.

### 4.2 Nuclear Hartree Product Representation Enhances Accuracy of Perturbative Corrections

The nuclear HP representation can also be used for calculating perturbative triples, such as electron–electron–electron triples and electron–electron–nucleus triples, which are denoted (T_eee_) and (T_een_), respectively. The equations for (T_eee_) do not directly involve the nuclei and thus are unchanged under the nuclear HP representation.^39,40^ The equations for (T_een_) are also unchanged because none of the terms can involve any nucleus besides the one in the triple excitation,^39,40^ but they are now calculated separately for each quantum nucleus. As previously these equations were provided specifically for the case of quantum protons,^39,40^ to match the convention used in this paper, terms involving electron–proton integrals should be multiplied by negative one and replaced with electron–nucleus integrals that include the charge of the nucleus implicitly.

Within the NEO framework, the proton affinity (PA) of species A can be calculated as

15 $$\mathrm{PA}\left(A\right)=E_{A}-E_{{\mathrm{AH}}^{+}}+\frac{5}{2}RT$$

where *E*_A_ is the energy of the unprotonated species and $E_{{\mathrm{AH}}^{+}}$ is the energy of the protonated species, *R* is the ideal gas constant, and *T* is the temperature. The ZPE of the proton added to A is incorporated by treating it quantum mechanically, and the other protons may also be treated quantum mechanically as long as they are treated consistently for both A and AH^+^ to include any ZPE changes for these protons. The ZPE contributions from the other nuclei are assumed to cancel out, as validated by earlier work.^37^ Proton affinities serve as a useful benchmark for NEO methods because they require accurate ZPEs.^28,37–39,73,74^ Table 2 shows calculations of percent error in correlation energy for species A and AH^+^ as well as the error in the calculated PA using various methods compared to the full electron–electron–nucleus triples method, NEO-CCSDT_een_ with the nuclear SD representation.^39^ The level of theory is the same as that used in the previous section.

**Table 2:** Percent Errors in Correlation Energy and Errors in PAs in eV Compared to NEO-CCSDT_een_ SD

molecule (A)	method	A corr. error (%)	AH^+^ corr. error (%)	PA error
CH_3_OH	NEO-MP2 SD	13.19	15.33	–0.338
	NEO-MP2 HP	8.09	9.55	–0.229
	NEO-CCSD	1.24	1.44	–0.032
	NEO-CCSD(T_een_) SD	0.73	0.83	–0.016
	NEO-CCSD(T_een_) HP	0.34	0.39	–0.009
CH_3_NH_2_	NEO-MP2 SD	16.69	18.80	–0.348
	NEO-MP2 HP	10.25	11.63	–0.226
	NEO-CCSD	1.50	1.66	–0.027
	NEO-CCSD(T_een_) SD	0.88	0.96	–0.013
	NEO-CCSD(T_een_) HP	0.40	0.45	–0.007
(H_2_O)_2_	NEO-MP2 SD	9.08	10.51	–0.271
	NEO-MP2 HP	5.24	6.19	–0.178
	NEO-CCSD	1.21	1.30	–0.019
	NEO-CCSD(T_een_) SD	0.71	0.71	–0.003
	NEO-CCSD(T_een_) HP	0.33	0.32	0.000
MUE	NEO-MP2 SD	12.99	14.88	0.319
	NEO-MP2 HP	7.86	9.12	0.211
	NEO-CCSD	1.32	1.47	0.026
	NEO-CCSD(T_een_) SD	0.77	0.83	0.010
	NEO-CCSD(T_een_) HP	0.36	0.39	0.006

Comparison of the proton affinities computed with the various methods provides insight into the impact of the nuclear HP representation. NEO-MP2 with the nuclear HP representation offers greatly improved results over the nuclear SD representation for both recovery of the correlation energy for A and AH^+^ as well as for the calculated PAs. For all these quantities, the mean unsigned error (MUE) is reduced by around a third. However, NEO-MP2 is still highly inaccurate for proton affinities, largely due to missing electron–proton correlation. NEO-CCSD recovers most of this missing correlation energy for both A and AH^+^ and greatly reduces the MUE of the PAs. The NEO-CCSD energies are nearly identical for the nuclear SD versus HP representations. The energy difference found with NEO-CCSD for SD versus HP were negligible and were determined to be from density fitting. For example, NEO-CCSD SD and HP energy calculations on H_2_O with the same setup as those in Table 2 agree to around 10^–6^ E_h_ when tightly converged. NEO-CCSD­(T_een_) with the nuclear SD representation decreases the MUEs by around 50% compared to NEO-CCSD. NEO-CCSD­(T_een_) with the nuclear HP representation is more accurate, decreasing the MUEs of the correlation energies by around another 50%. These trends in the MUEs are observed for all three molecules, showing that the improvements between methods are consistent.

To understand these trends, we first consider the non-perturbative methods, such as NEO-CCSD and NEO-CCSDT_een_. For a single quantum nucleus, the second-quantization Hamiltonian is the same within both representations, and the virtual orbitals are related by a unitary transformation. Because the CC energy is invariant to unitary transformations of the virtual orbitals,^75^ the NEO-CC energies are unchanged. For multiple quantum fermionic nuclei, the additional difference between the nuclear HP and SD representations is nuclear exchange, which is expected to be negligible for the systems studied here. Thus, the nuclear SD and HP representation energies for NEO-CC methods using full excitations are expected to agree for multiple quantum nuclei as well. Our results with the NEO-CCSD method shown in Table 2 are consistent with this analysis.

Next, we consider the perturbative methods. As discussed previously for the case of a single quantum proton, the zeroth-order Hamiltonian and perturbation in NEO perturbative methods are changed when self-Coulomb and self-exchange terms are included in the Fock operator, allowing for differences in the perturbative energy corrections.^34,39^ This previous work showed that the perturbative corrections are more accurate when the self-Coulomb and self-exchange terms are not included in the Fock operator. This behavior has been observed for NEO-MP2 ^34^ as well as NEO-CCSD­(T_een_).^39^ Because the nuclear SD representation includes self-Coulomb and self-exchange terms in the Fock operator, but the nuclear HP representation does not include these terms, the perturbative energy corrections for multiple quantum protons can be improved in a similar manner by using the nuclear HP representation. The results in Table 2 show this improvement for NEO-CCSD­(T_een_) when the nuclear HP representation is used. The improved accuracy is most likely due to using a zeroth-order Hamiltonian associated with more physically reasonable virtual orbitals and virtual orbital energies, as shown in Section S3 and Figure S1 of the Supporting Information. Moreover, the difference in the energies computed with NEO-CCSD­(T_een_) for the nuclear HP and SD representations does not appear to be related to orbital relaxation in NEO-CCSD, as this difference is also observed for the NEO-CCD­(T_een_) method, which does not include orbital relaxation (Table S3). Orbital relaxation in NEO-CCSD also does not appear to change the qualitative differences between the virtual orbitals for the two nuclear representations (Figure S2).

Thus, NEO-CCSD­(T_een_) with the nuclear HP representation is a highly accurate approximation of NEO-CCSDT_een_, even more accurate than NEO-CCSD­(T_een_) with the nuclear SD representation, and at a significantly reduced cost. The form of the perturbative triple electronic excitations is unchanged with the nuclear HP representation, but inclusion of these excitations leads to the NEO-CCSD­(T) method, the equivalent of the “gold standard” in conventional electronic structure theory for recovering dynamical correlation.

### 4.3 Interaction Energies for Systems with Multiple Hydrogen Bonds

To demonstrate the capabilities of NEO-CCSD­(T) with the nuclear HP representation, we calculated interaction energies for systems with multiple hydrogen bonds. To further extend the largest feasible system size for NEO-CCSD­(T), we also explored electronic FNO basis set truncation.^58^ The necessary equations for the NEO-MP2 electronic one-particle density matrix have been provided in other work.^35,59^ Here, truncation of the electronic FNO basis was performed to retain at least 99.0% of the occupation of the virtual–virtual block of the diagonalized NEO-MP2 electronic one-particle density matrix. Additionally, a correction was applied to account for the discarded orbitals by including the difference between the NEO-MP2 correlation energies in the untruncated and truncated FNO basis sets. This level of truncation was found to provide very large speedups because only 35–55% of the virtual orbitals were kept, depending on the system and the electronic atomic orbital basis set size. Additionally, FNO truncation dramatically reduces memory usage, which scales as $O\left(ov^{3}\right)$ for this implementation of NEO-CCSD­(T), where *o* is the number of electronic occupied orbitals and *v* is the number of electronic virtual orbitals retained after truncation.

The calculated interaction energies with and without FNO basis set truncation are given in Table 3. The atomic basis sets are the same as those used in the previous subsection. The dimer geometries were optimized at the conventional MP2/aug-cc-pVTZ level of theory. The geometries used for calculating the interaction energies are shown in Figure 1. As NEO-CCSD­(T) proton densities are not available, the proton densities in all figures shown herein were computed with NEO-DFT/*ω*B97X-D/epc17-2/def2-SVP/PB4-F2 ^28,68,76,77^ for visualization purposes. The monomer geometries were fixed to their geometries in the optimized dimer. Because these calculations do not include monomer relaxation, correction for basis set superposition error, heavy-atom ZPE, and thermal energy corrections, they are not directly comparable to experimental binding energies and likely overestimate the binding energies. Typically, interaction energies represent only one component of a calculated binding energy for comparison to experiment. Thus, these calculations are only meant to serve as an example of the kinds of systems that are accessible with NEO-CCSD­(T).

**Figure 1:**
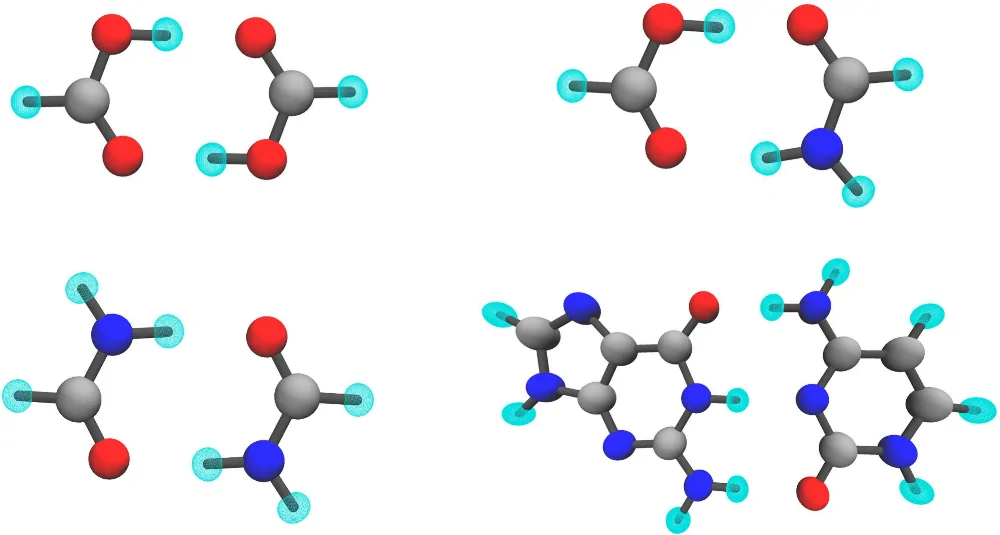
Formic acid dimer (upper left), formic acid/formamide dimer (upper right), formamide dimer (lower left), and guanine/cytosine dimer (lower right) geometries used for calculating the NEO-CCSD­(T) and CCSD­(T) interaction energies. All protons are treated quantum mechanically for the NEO calculations, and their proton densities are shown with cyan meshes.

**Table 3:** Computed Interaction Energies Using NEO-CCSD­(T) with and without FNO Truncation and Using Conventional CCSD­(T) with FNO Truncation in kcal/mol

molecule	FNO	no FNO	conv./FNO
formic acid dimer	21.25	21.24	19.57
formic acid/formamide dimer	20.33	20.28	18.94
formamide dimer	17.96	17.88	16.72
guanine/cytosine dimer	35.69	ND^a^	33.60

a ND indicates not determined.

The calculated interaction energies using the FNO truncation are within 0.1 kcal/mol of the values calculated without FNO truncation. For the guanine/cytosine dimer, the interaction energy was only calculated using the FNO truncation due to computational expense. We also calculated the interaction energies using conventional CCSD­(T) with the same FNO procedure (Table 3). The differences between these conventional interaction energies and those calculated using NEO-CCSD­(T) illustrate the importance of including the proton ZPE and delocalization effects.

As examples of systems with even more quantum protons, we computed the energy of water clusters containing 10 and 12 water molecules using NEO-CCSD­(T) with the nuclear HP representation and FNO truncation. The geometries were optimized at the conventional MP2/aug-cc-pVTZ level of theory. The geometries used for these calculations are shown in Figure 2. We were able to converge the NEO-CCSD *t*-amplitude equations in 10 iterations for (H_2_O)_10_ and 13 iterations for (H_2_O)_12_. The total calculation required only 251 CPU hours for (H_2_O)_10_ and 853 CPU hours for (H_2_O)_12_ on a computer using 3.1 GHz Intel Cascade Lake processors. These results illustrate that NEO-CCSD­(T) with the nuclear HP representation and FNO truncation can be used to obtain accurate results on medium-sized systems with many quantum mechanically treated nuclei.

**Figure 2:**
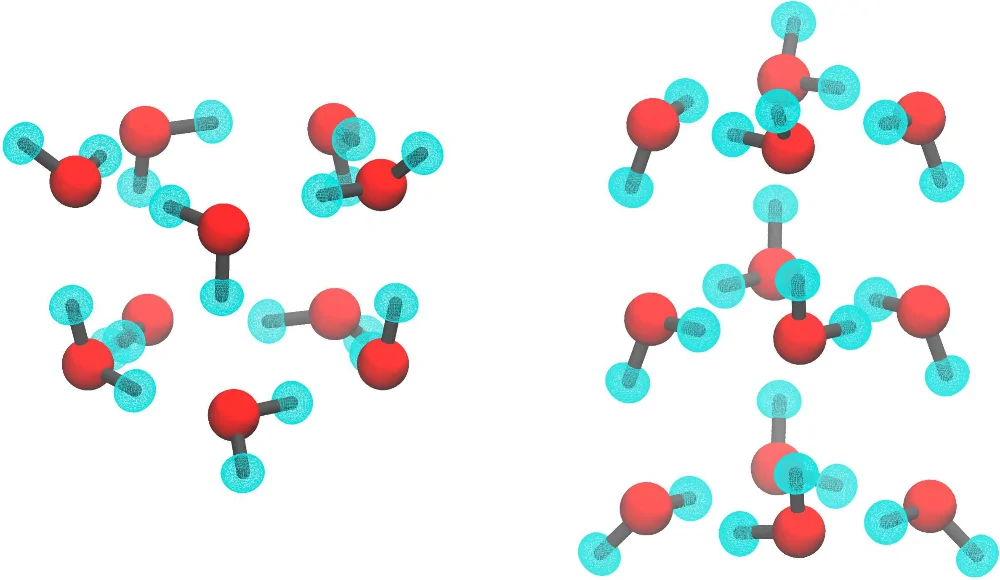
Antiparallel (H_2_O)_10_ geometry (left) and (H_2_O)_12_ geometry (right) used for the NEO-CCSD­(T) calculations. All protons are treated quantum mechanically, and their proton densities are shown with cyan meshes.

### 4.4 Geometric Isotope Effects

Isotopic substitution, such as protium (H) for deuterium (D), can affect the structure of molecules and materials due to nuclear quantum effects such as nuclear delocalization and ZPE, resulting in geometric isotope effects (GIEs). In some cases, these GIEs can affect other properties. A notable example is the large isotope effect on the phase transition temperature of hydrogen-bonded ferroelectric crystals such as potassium dihydrogen phosphate.^78,79^ Often, hydrogen-bonded systems experience an elongation of the donor–acceptor distance upon deuteration,^80–82^ although in some cases, the donor–acceptor distance is shortened upon deuteration. This varied behavior is thought to arise from a competition between the ZPE and delocalization effects on the bending modes versus the stretching modes of a hydrogen bond.^83–85^ Herein, we will use NEO-CCSD­(T) to compute GIEs associated with heavy-atom distances, such as the donor–acceptor distance in a hydrogen bond.

Geometry optimizations with conventional electronic structure methods cannot be used to compute GIEs because the geometries do not depend on the masses. Adding the ZPE computed from normal modes based on the harmonic approximation as a function of a specific heavy-atom distance cannot be used to compute GIEs because the normal mode analysis is only valid at minimum-energy geometries. Second-order vibrational perturbation theory (VPT2) can be used to compute GIEs by first performing a normal mode analysis and then computing anharmonic corrections that can be used to obtain vibrationally averaged structures.^86^ However, this procedure becomes expensive for large molecules and has limitations due to the perturbative nature of the corrections. NEO-CCSD­(T) provides an accurate and efficient way to include the anharmonic ZPE of protons and deuterons at any geometry without requiring a perturbative treatment of the anharmonicity. Thus, a NEO-CCSD­(T) geometry optimization can be performed to obtain GIEs associated with the substitution of H with D. The nuclear HP representation extends the applicability of this approach to larger systems with more quantum nuclei.

NEO methods and related multicomponent methods have been used to study GIEs in the past.^19,56,87–93^ NEO-CCSD and NEO-CCSD­(T) have previously been used to calculate an optimized geometry of FHF^–^ compared to an accurate reference.^40,48^ However, NEO-CCSD­(T) has not been used to optimize geometries with multiple quantum protons or to compute GIEs. To test the capabilities of NEO-CCSD­(T) for predicting accurate GIEs, we benchmarked our results for the FHF^–^ and FDF^–^ systems against the FGH/CCSD­(T) calculations discussed in Section 3. The FGH method is numerically exact at a given level of electronic structure theory for electronically adiabatic systems. The NEO-CCSD­(T) calculations were performed using the aug-cc-pVTZ, aug-cc-pVQZ, and aug-cc-pV5Z electronic basis sets, where the quantum nuclear basis function center was positioned at the midpoint between the two F atoms. The NEO-CCSD­(T)/aug-cc-pV5Z results are the most comparable to the FGH/CCSD­(T) results, which were obtained by adding the FGH ZPE to the CCSD­(T)/aug-cc-pV5Z electronic energy.

The FHF^–^ and FDF^–^ energies as a function of F–F distance calculated with NEO-CCSD­(T)/aug-cc-pV5Z and FGH/CCSD­(T) are shown in Figure 3. The distances corresponding to the minimum energy at each level of theory are given in Table 4. The GIE for FHF^–^ has been studied computationally in the past by exploiting the limited nuclear degrees of freedom, predicting a decrease in F–F distance of 0.0058 Å upon deuteration,^94^ a very similar result as the FGH/CCSD­(T) reference value obtained here (Table 4).

**Figure 3:**
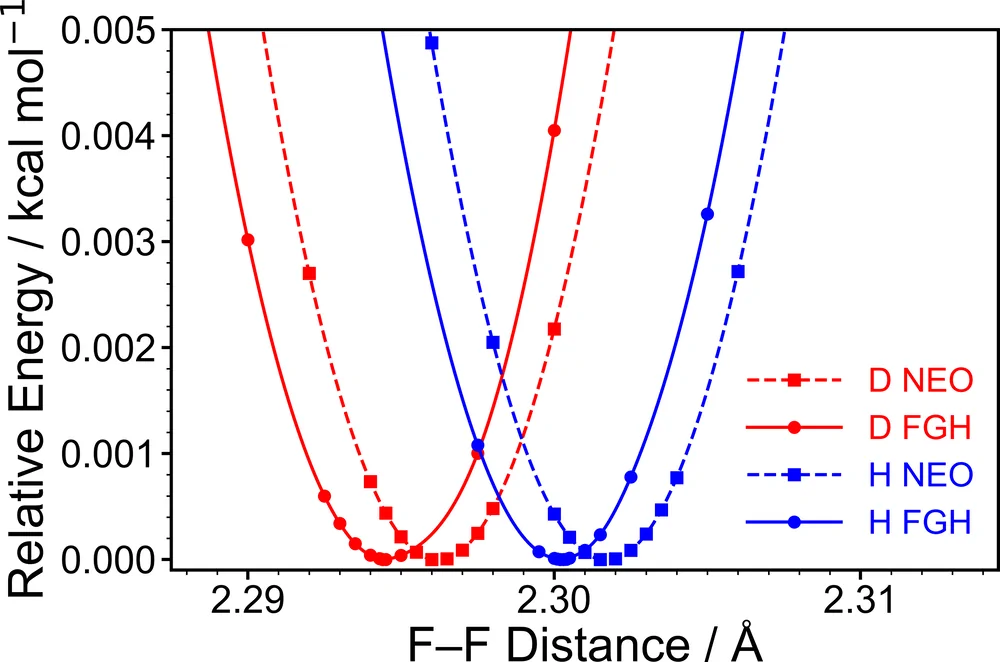
Relative energies of FHF^–^ (blue) and FDF^–^ (red) at various F–F distances computed with NEO-CCSD­(T) (squares, dashed line spline fit) and FGH/CCSD­(T) reference (circles, solid line spline fit).

**Table 4:** Optimized F–F Distances for FHF^–^ and FDF^–^ and Geometric Isotope Effects (GIEs) in Angstroms Computed with NEO-CCSD­(T) Using Various Electronic Basis Sets with and without FNO Truncation as well as the Reference FGH/CCSD­(T) Values

electronic basis	FNO	H	D	GIE
aug-cc-pVTZ	Yes	2.3039	2.2974	–0.0065
	No	2.3067	2.2994	–0.0073
aug-cc-pVQZ	Yes	2.3017	2.2957	–0.0060
	No	2.3040	2.2979	–0.0061
aug-cc-pV5Z	Yes	2.3000	2.2946	–0.0054
	No	2.3017	2.2962	–0.0055
FGH/CCSD(T) (reference)		2.3003	2.2944	–0.0059

NEO-CCSD­(T) predicts very similar optimized F–F distances as does the reference FGH/CCSD­(T) method for both FHF^–^ and FDF^–^, and the predicted GIE is within 0.0005 Å. Furthermore, the shapes of the one-dimensional potential energy surfaces for both FHF^–^ and FDF^–^ are very similar for NEO-CCSD­(T) and FGH/CCSD­(T), except that the NEO-CCSD­(T) minima are at slightly longer F–F distances. The similarity of the NEO-CCSD­(T) results to the grid-based reference shows that NEO-CCSD­(T) is capable of predicting accurate GIEs through only two geometry optimizations, one with H and one with D. In comparison, for cases with no symmetry, the FGH/CCSD­(T) approach requires tens of thousands of single-point energy calculations for each heavy-atom distance sampled for both isotopes. Furthermore, NEO-CCSD­(T) can be applied to systems with many quantum mechanically treated nuclei, whereas it is computationally impractical to use this FGH approach for more than one quantum nucleus moving in three dimensions. Path integral approaches allow the calculation of GIEs for systems with multiple quantum nuclei, but they are also expensive, particularly at very low temperatures to obtain optimized geometries.^95,96^ Thus, NEO-CCSD­(T) with the nuclear HP representation represents an accurate and practical means for studying GIEs in systems with many quantum nuclei.

Before calculating GIEs on larger systems with NEO-CCSD­(T), we examine the necessity for large electronic basis sets and examine the utility of FNO truncation. The optimal F–F distances and GIEs for FHF^–^/FDF^–^ calculated with NEO-CCSD­(T) were found to be dependent on the electronic basis set, as shown in Table 4. Also shown are the optimal F–F distances and GIEs calculated with the FNO truncation procedure outlined above. For calculations without FNO truncation, the minimum-energy F–F distances change slightly more than the GIEs with the electronic basis set. The GIE appears to be nearly converged with the largest electronic basis set tested. Calculations using FNO truncation predict similar GIEs as predicted by the untruncated calculations. In particular, for all but the smallest electronic basis set, the predicted GIE with FNO truncation is within 0.0001 Å of the value obtained from the untruncated calculation. This observation indicates that FNO truncation in conjunction with sufficient electronic basis sets can efficiently predict accurate GIEs. The number of electronic virtual orbitals retained in the FNO calculations was 83 for the aug-cc-pVQZ basis set and 93 for the aug-cc-pV5Z basis set. Thus, the aug-cc-pV5Z NEO-CCSD­(T) procedure was only slightly more expensive than the aug-cc-pVQZ NEO-CCSD­(T) procedure for the *t*-amplitude equations and perturbative correction.

Next, we calculate the GIEs for a water dimer and a series of small HF clusters. The optimized geometries for (H_2_O)_2_ and (HF)_5_ are depicted in Figure 4. These geometry optimizations were performed using numerical gradients, taking advantage of symmetry. The (HF)_3_, (HF)_4_, and (HF)_5_ rings are assumed to have *C*_3*h*_, *C*_4*h*_, and *C*_5*h*_ symmetry, respectively, which are likely the symmetries of the global minimum energies based on the geometries optimized with conventional electronic structure theory.^97^ Table 5 provides the optimized closest heavy-atom distances and GIEs for a variety of systems at the FNO-NEO-CCSD­(T)/aug-cc-pVQZ level of theory. Also given are the optimized heavy-atom distances and GIEs when only the hydrogen-bonding hydrogen nucleus is treated quantum mechanically for the (HF)_2_ and (H_2_O)_2_ dimers. When all hydrogen nuclei are treated quantum mechanically, the predicted GIEs are different, showing that nuclei that are not directly involved in the hydrogen bond can impact the GIEs, presumably through their delocalization and ZPEs. For all these systems, the heavy-atom distances are lengthened upon deuteration, whereas for FHF^–^ the heavy atom distance was shortened. These results show that NEO can capture both positive and negative GIEs. For the HF clusters, the GIE increases in magnitude as the size of the cluster increases, possibly due to the increase in hydrogen-bonding cooperativity.

**Figure 4:**
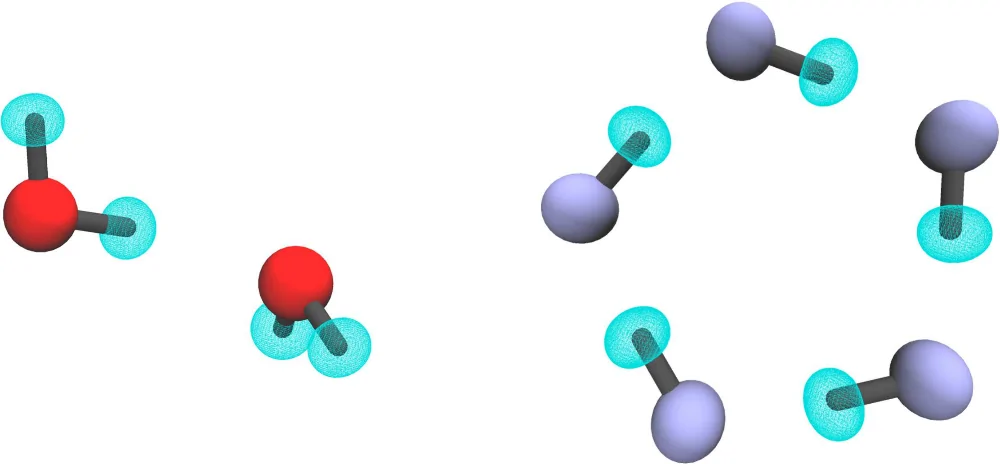
Water dimer and (HF)_5_ optimized geometries calculated with NEO-CCSD­(T). All protons are treated quantum mechanically, and their proton densities are shown with cyan meshes.

**Table 5:** Optimized NEO-CCSD­(T) O–O or Shortest F–F Distances and Geometric Isotope Effects (GIEs) in Angstroms

system	H	D	GIE
(H_2_O)_2_ ^a^	2.9045	2.9071	0.0026
(H_2_O)_2_	2.8997	2.9025	0.0028
(HF)_2_ ^a^	2.7292	2.7297	0.0005
(HF)_2_	2.7226	2.7261	0.0036
(HF)_3_	2.5657	2.5767	0.0110
(HF)_4_	2.4661	2.4839	0.0179
(HF)_5_	2.4283	2.4494	0.0211

a Only the hydrogen-bonded proton was treated quantum mechanically for these two cases.

## 5 Conclusions

This work presents a formulation of NEO-CCSD with the nuclear Hartree product representation for an arbitrary number of quantum mechanically treated nuclei. Straightforward modifications of the nuclear Slater determinant formulation to obtain the NEO-CCSD­(T) triple excitations correction are also presented. This NEO-CCSD­(T) method is applied to calculations that treat all hydrogen or deuterium nuclei quantum mechanically. We show that the nuclear Hartree product representation significantly decreases the number of iterations required for the iterative convergence of the NEO-CCSD *t*-amplitude equations, leading to substantial computational savings. These NEO-CC calculations also benefit from the improved iterative convergence of NEO-HF within the nuclear Hartree product representation.^54^ Furthermore, our calculations show that perturbative corrections in methods such as NEO-MP2 and NEO-CCSD­(T) are significantly more accurate with the nuclear Hartree product representation compared to the nuclear Slater determinant representation. This greater accuracy of the perturbative corrections is most likely due to more physically reasonable virtual orbitals. An example of this improvement is that the errors in the correlation energy and the absolute errors in the proton affinities compared to the full triples reference method are reduced by around 50% for the systems studied.

To showcase the broad applicability of this NEO-CCSD­(T) formulation, we used it to calculate interaction energies for systems containing multiple hydrogen bonds. An FNO basis set truncation procedure was implemented to further extend the attainable system size. NEO-CCSD­(T) was used to calculate the interaction energy of a guanine/cytosine dimer with all protons treated quantum mechanically and the energies of water clusters composed of 10 and 12 water molecules with 20 and 24 quantum protons, respectively. Furthermore, due to the high accuracy and efficiency offered by this NEO-CCSD­(T) formulation, we used it to compute geometric isotope effects. NEO-CCSD­(T) was found to agree quantitatively with the geometric isotope effects obtained with a numerically exact grid-based reference for FHF^–^ and FDF^–^ both with and without the FNO truncation procedure. Using the FNO truncation, NEO-CCSD­(T) was used to calculate geometric isotope effects in systems containing multiple hydrogen bonds, including a water dimer and HF clusters composed of up to five HF molecules. NEO-CCSD­(T) was found to be able to predict both positive and negative geometric isotope effects. The heavy-atom distance decreases upon deuteration for FHF^–^ and increases upon deuteration for the water dimer and HF clusters studied. Moreover, the magnitude of this distance change increases as the cluster size increases for the HF clusters.

The formulation of NEO-CC provided here will also be useful for calculations with multiple types of quantum nuclei, such as partially deuterated molecules or full quantum treatments. Additionally, this formulation is also expected to be useful for the development of constrained NEO (CNEO)^20,25,93^ CC or MP2 methods, where quantum nuclei must be treated as distinguishable particles. Overall, NEO-CCSD­(T) with the nuclear Hartree product representation provides an efficient, accurate, and flexible method for incorporating nuclear quantum effects into ground-state calculations and may serve as a benchmark for the development of lower-level NEO methods.

## Supplementary Material



## Data Availability

The data that support the findings of this study are available in the published article, the Supporting Information, and Zenodo at 10.5281/zenodo.21630818.

## Notes

Discussion of self-Coulomb and self-exchange; Additional comparisons of NEO-CCSD iterative convergence; Virtual orbital comparisons; Perturbative triples correction when excluding single excitations; Nuclear HP NEO-CCSD programmable *t*-amplitude and energy equations; Setup for obtaining the programmable equations when including double nuclear excitations (PDF)

## References

[1] Webb S. P., Iordanov T., Hammes-Schiffer S. (2002). Multiconfigurational nuclear-electronic
orbital approach: Incorporation of nuclear quantum effects in electronic
structure calculations. J. Chem. Phys..

[2] Pavošević F., Culpitt T., Hammes-Schiffer S. (2020). Multicomponent Quantum Chemistry:
Integrating Electronic and Nuclear Quantum Effects via the Nuclear–Electronic
Orbital Method. Chem. Rev..

[3] Hammes-Schiffer S. (2021). Nuclear–electronic
orbital methods: Foundations and prospects. J. Chem. Phys..

[4] Yang Y., Culpitt T., Hammes-Schiffer S. (2018). Multicomponent
Time-Dependent Density
Functional Theory: Proton and Electron Excitation Energies. J. Phys. Chem. Lett..

[5] Garner S. M., Upadhyay S., Li X., Hammes-Schiffer S. (2024). Nuclear–Electronic
Orbital Time-Dependent Configuration Interaction Method. J. Phys. Chem. Lett..

[6] Garner S. M., Upadhyay S., Li X., Hammes-Schiffer S. (2025). Time-resolved
vibronic spectra with nuclear–electronic orbital time-dependent
configuration interaction. J. Chem. Phys..

[7] Aieta C., Garner S. M., Liu A., Li X., Hammes-Schiffer S. (2026). Nuclear–electronic
orbital quasiclassical trajectory method for vibrational spectroscopy. J. Chem. Phys..

[8] Schneider P.
E., Tao Z., Pavošević F., Epifanovsky E., Feng X., Hammes-Schiffer S. (2021). Transition states, reaction paths,
and thermochemistry using the nuclear–electronic orbital analytic
Hessian. J. Chem. Phys..

[9] Zhao L., Wildman A., Pavošević F., Tully J. C., Hammes-Schiffer S., Li X. (2021). Excited State Intramolecular Proton
Transfer with Nuclear-Electronic Orbital Ehrenfest Dynamics. J. Phys. Chem. Lett..

[10] Dickinson J. A., Hammes-Schiffer S. (2024). Nonadiabatic Hydrogen Tunneling Dynamics
for Multiple
Proton Transfer Processes with Generalized Nuclear-Electronic Orbital
Multistate Density Functional Theory. J. Chem.
Theory Comput..

[11] Dickinson J. A., Chow M., Paenurk E., Hammes-Schiffer S. (2026). Extended Lagrangian
molecular dynamics on vibronic surfaces in the nuclear–electronic
orbital framework. J. Chem. Phys..

[12] Hasecke L., Breitenbach M., Gimferrer M., Oswald R., Mata R. A. (2025). Addressing
Anharmonic Effects with Density-Fitted Multicomponent Density Functional
Theory. J. Phys. Chem. A.

[13] Yoo J. M., Chow M., Paenurk E., Hammes-Schiffer S. (2025). Reliable p*K*
_a_ Prediction
through Efficient Incorporation
of Anharmonicity within the Nuclear–Electronic Orbital Framework. J. Am. Chem. Soc..

[14] Chow M., Reinhardt C. R., Hammes-Schiffer S. (2024). Nuclear Quantum Effects in Quantum
Mechanical/Molecular Mechanical Free Energy Simulations of Ribonucleotide
Reductase. J. Am. Chem. Soc..

[15] Smith L. E., Settembri P., Cucciari A., Boeri L., Profeta G., Hammes-Schiffer S. (2026). Capturing
nuclear quantum effects in high-pressure
superconducting hydrides and ice with nuclear–electronic orbital
theory. Proc. Natl. Acad. Sci. U.S.A..

[16] Thomas I. L. (1969). Protonic
Structure of Molecules. I. Ammonia Molecules. Phys. Rev..

[17] Ishimoto T., Tachikawa M., Nagashima U. (2009). Review of multicomponent molecular
orbital method for direct treatment of nuclear quantum effect. Int. J. Quantum Chem..

[18] Kreibich T., Gross E. K. U. (2001). Multicomponent Density-Functional Theory for Electrons
and Nuclei. Phys. Rev. Lett..

[19] Reyes A., Moncada F., Charry J. (2019). The any particle
molecular orbital
approach: A short review of the theory and applications. Int. J. Quantum Chem..

[20] Xu X., Yang Y. (2020). Constrained nuclear-electronic orbital density functional theory:
Energy surfaces with nuclear quantum effects. J. Chem. Phys..

[21] Brorsen K. R. (2020). Quantifying
Multireference Character in Multicomponent Systems with Heat-Bath
Configuration Interaction. J. Chem. Theory Comput..

[22] Muolo A., Baiardi A., Feldmann R., Reiher M. (2020). Nuclear-electronic
all-particle density matrix renormalization group. J. Chem. Phys..

[23] White A. F., Gao Y., Minnich A. J., Chan G. K.-L. (2020). A coupled cluster framework for electrons
and phonons. J. Chem. Phys..

[24] Hasecke L., Mata R. A. (2023). Nuclear Quantum Effects Made Accessible: Local Density
Fitting in Multicomponent Methods. J. Chem.
Theory Comput..

[25] Tucker G. B., Brorsen K. R. (2026). Constrained nuclear–electronic
orbital second-order
Møller–Plesset perturbation theory. J. Chem. Phys..

[26] Pak M. V., Chakraborty A., Hammes-Schiffer S. (2007). Density Functional Theory Treatment
of Electron Correlation in the Nuclear–Electronic Orbital Approach. J. Phys. Chem. A.

[27] Yang Y., Brorsen K. R., Culpitt T., Pak M. V., Hammes-Schiffer S. (2017). Development
of a practical multicomponent density functional for electron-proton
correlation to produce accurate proton densities. J. Chem. Phys..

[28] Brorsen K. R., Yang Y., Hammes-Schiffer S. (2017). Multicomponent
Density Functional
Theory: Impact of Nuclear Quantum Effects on Proton Affinities and
Geometries. J. Phys. Chem. Lett..

[29] Tao Z., Yang Y., Hammes-Schiffer S. (2019). Multicomponent
density functional
theory: Including the density gradient in the electron-proton correlation
functional for hydrogen and deuterium. J. Chem.
Phys..

[30] Yu Q., Hammes-Schiffer S. (2020). Nuclear-Electronic
Orbital Multistate Density Functional
Theory. J. Phys. Chem. Lett..

[31] Dickinson J. A., Yu Q., Hammes-Schiffer S. (2023). Generalized
Nuclear-Electronic Orbital
Multistate Density Functional Theory for Multiple Proton Transfer
Processes. J. Phys. Chem. Lett..

[32] Culpitt T., Yang Y., Pavošević F., Tao Z., Hammes-Schiffer S. (2019). Enhancing the applicability of multicomponent
time-dependent
density functional theory. J. Chem. Phys..

[33] Nakai H., Sodeyama K. (2003). Many-body effects in
nonadiabatic molecular theory
for simultaneous determination of nuclear and electronic wave functions: *Ab initio* NOMO/MBPT and CC methods. J. Chem. Phys..

[34] Swalina C., Pak M. V., Hammes-Schiffer S. (2005). Alternative formulation of many-body
perturbation theory for electron–proton correlation. Chem. Phys. Lett..

[35] Pavošević F., Rousseau B. J. G., Hammes-Schiffer S. (2020). Multicomponent
Orbital-Optimized
Perturbation Theory Methods: Approaching Coupled Cluster Accuracy
at Lower Cost. J. Phys. Chem. Lett..

[36] Fajen O. J., Brorsen K. R. (2020). Separation of electron–electron
and electron–proton
correlation in multicomponent orbital-optimized perturbation theory. J. Chem. Phys..

[37] Pavošević F., Culpitt T., Hammes-Schiffer S. (2019). Multicomponent Coupled Cluster Singles
and Doubles Theory within the Nuclear-Electronic Orbital Framework. J. Chem. Theory Comput..

[38] Pavošević F., Hammes-Schiffer S. (2022). Triple electron–electron–proton
excitations
and second-order approximations in nuclear–electronic orbital
coupled cluster methods. J. Chem. Phys..

[39] Goudy R. J., Pavošević F., Hammes-Schiffer S. (2025). Triple excitations
in nuclear–electronic orbital coupled cluster theory for multiple
quantum protons. J. Chem. Phys..

[40] Fowler D., Brorsen K. R. (2022). (T) Correction for
Multicomponent Coupled-Cluster Theory
for a Single Quantum Proton. J. Chem. Theory
Comput..

[41] Malbon C. L., Hammes-Schiffer S. (2025). Nuclear–Electronic Orbital Multireference Configuration
Interaction for Ground and Excited Vibronic States and Fundamental
Insights into Multicomponent Basis Sets. J.
Chem. Theory Comput..

[42] Stein R. J., Malbon C. L., Hammes-Schiffer S. (2025). Computing
Hydrogen Tunneling Splittings
with Nuclear–Electronic Orbital Multireference Configuration
Interaction. J. Phys. Chem. Lett..

[43] Fetherolf J. H., Pavošević F., Hammes-Schiffer S. (2026). Nuclear–electronic
orbital second-order coupled cluster for excited states. J. Chem. Phys..

[44] Pavošević F., Hammes-Schiffer S. (2019). Multicomponent equation-of-motion coupled cluster singles
and doubles: Theory and calculation of excitation energies for positronium
hydride. J. Chem. Phys..

[45] Pavošević F., Tao Z., Culpitt T., Zhao L., Li X., Hammes-Schiffer S. (2020). Frequency
and Time Domain Nuclear–Electronic Orbital Equation-of-Motion
Coupled Cluster Methods: Combination Bands and Electronic–Protonic
Double Excitations. J. Phys. Chem. Lett..

[46] Pavošević F., Hammes-Schiffer S. (2021). Multicomponent
Unitary Coupled Cluster and Equation-of-Motion
for Quantum Computation. J. Chem. Theory Comput..

[47] Lyakh D. I., Musiał M., Lotrich V. F., Bartlett R. J. (2012). Multireference Nature
of Chemistry: The Coupled-Cluster View. Chem.
Rev..

[48] Pavošević F., Hammes-Schiffer S. (2019). Multicomponent coupled cluster singles and doubles
and Brueckner doubles methods: Proton densities and energies. J. Chem. Phys..

[49] Pavošević F., Tao Z., Hammes-Schiffer S. (2021). Multicomponent Coupled Cluster Singles
and Doubles with Density Fitting: Protonated Water Tetramers with
Quantized Protons. J. Phys. Chem. Lett..

[50] Lambros E., Fetherolf J. H., Hammes-Schiffer S., Li X. (2024). A Many-Body Perspective
of Nuclear Quantum Effects in Aqueous Clusters. J. Phys. Chem. Lett..

[51] Karton A. (2016). How large
are post-CCSD­(T) contributions to the total atomization energies of
medium-sized alkanes?. Chem. Phys. Lett..

[52] Smith D. G. A., Jankowski P., Slawik M., Witek H. A., Patkowski K. (2014). Basis Set
Convergence of the Post-CCSD­(T) Contribution to Noncovalent Interaction
Energies. J. Chem. Theory Comput..

[53] Raghavachari K., Trucks G. W., Pople J. A., Head-Gordon M. (1989). A fifth-order
perturbation comparison of electron correlation theories. Chem. Phys. Lett..

[54] Chow M., Paenurk E., Hammes-Schiffer S. (2026). Efficient
and robust optimization
of nuclear and electronic orbitals within the nuclear Hartree product
representation. J. Chem. Phys..

[55] Auer B., Hammes-Schiffer S. (2010). Localized
Hartree product treatment of multiple protons
in the nuclear-electronic orbital framework. J. Chem. Phys..

[56] Culpitt T., Chen Z., Pavošević F., Yang Y. (2025). Constrained
Nuclear-Electronic Orbital Theory for Quantum Computation. J. Chem. Theory Comput..

[57] Tucker G. B., Brorsen K. R. (2026). An Analytic Nuclear Energy Gradient
for Multicomponent
CCSD. J. Chem. Theory Comput..

[58] Taube A. G., Bartlett R. J. (2005). Frozen Natural Orbitals:
Systematic Basis Set Truncation
for Coupled-Cluster Theory. Collect. Czech.
Chem. Commun..

[59] Nykänen A., Miller A., Talarico W., Knecht S., Kovyrshin A., Skogh M., Tornberg L., Broo A., Mensa S., Symons B. C. B. (2023). Toward Accurate Post-Born–Oppenheimer
Molecular Simulations on Quantum Computers: An Adaptive Variational
Eigensolver with Nuclear-Electronic Frozen Natural Orbitals. J. Chem. Theory Comput..

[60] Shavitt, I. ; Bartlett, R. J. Many-body methods in chemistry and physics: MBPT and coupled-cluster theory. Cambridge Molecular Science; Cambridge University Press: Cambridge; New York, 2009; OCLC: ocn297147115.

[61] White, A. Wick v0.9.0. https://github.com/awhite862/wick, 2023.

[62] Epifanovsky E., Gilbert A. T. B., Feng X., Lee J., Mao Y., Mardirossian N., Pokhilko P., White A. F., Coons M. P., Dempwolff A. L. (2021). Software for the frontiers of quantum chemistry:
An overview of developments in the Q-Chem 5 package. J. Chem. Phys..

[63] Dunning T.
H. (1989). Gaussian
basis sets for use in correlated molecular calculations. I. The atoms
boron through neon and hydrogen. J. Chem. Phys..

[64] Van
Mourik T., Wilson A. K., Dunning T. H. (1999). Benchmark calculations with correlated molecular wavefunctions. XIII.
Potential energy curves for He2, Ne2 and Ar2 using correlation consistent
basis sets through augmented sextuple *ζ*. Mol. Phys..

[65] Weigend F., Häser M., Patzelt H., Ahlrichs R. (1998). RI-MP2: optimized
auxiliary
basis sets and demonstration of efficiency. Chem. Phys. Lett..

[66] Weigend F., Köhn A., Hättig C. (2002). Efficient
use of the correlation
consistent basis sets in resolution of the identity MP2 calculations. J. Chem. Phys..

[67] Hättig C. (2005). Optimization
of auxiliary basis sets for RI-MP2 and RI-CC2 calculations: Core–valence
and quintuple-zeta basis sets for H to Ar and QZVPP basis sets for
Li to Kr. Phys. Chem. Chem. Phys..

[68] Yu Q., Pavošević F., Hammes-Schiffer S. (2020). Development
of nuclear basis sets for multicomponent quantum chemistry methods. J. Chem. Phys..

[69] Helgaker, T. ; Jørgensen, P. ; Olsen, J. Molecular Electronic-Structure Theory; John Wiley & Sons, Ltd, 2000; pp 648–723, Section: 13 _eprint: https://onlinelibrary.wiley.com/doi/pdf/10.1002/9781119019572.ch13.

[70] Marston C. C., Balint-Kurti G. G. (1989). The Fourier
grid Hamiltonian method for bound state
eigenvalues and eigenfunctions. J. Chem. Phys..

[71] Webb S. P., Hammes-Schiffer S. (2000). Fourier grid
Hamiltonian multiconfigurational self-consistent-field:
A method to calculate multidimensional hydrogen vibrational wavefunctions. J. Chem. Phys..

[72] Heindel J. P., Yu Q., Bowman J. M., Xantheas S. S. (2018). Benchmark Electronic Structure Calculations
for H3O+(H2O)­n, n = 0–5, Clusters and Tests of an Existing
1,2,3-Body Potential Energy Surface with a New 4-Body Correction. J. Chem. Theory Comput..

[73] Díaz-Tinoco M., Romero J., Ortiz J. V., Reyes A., Flores-Moreno R. (2013). A generalized
any-particle propagator theory: Prediction of proton affinities and
acidity properties with the proton propagator. J. Chem. Phys..

[74] Fetherolf J. H., Pavošević F., Tao Z., Hammes-Schiffer S. (2022). Multicomponent
Orbital-Optimized Perturbation Theory with Density Fitting: Anharmonic
Zero-Point Energies in Protonated Water Clusters. J. Phys. Chem. Lett..

[75] Jayatilaka D., Handy N. C. (1992). Implications of unitary invariance
for gradient theory. Int. J. Quantum Chem..

[76] Chai J.-D., Head-Gordon M. (2008). Long-range
corrected hybrid density functionals with
damped atom–atom dispersion corrections. Phys. Chem. Chem. Phys..

[77] Weigend F., Ahlrichs R. (2005). Balanced basis sets
of split valence, triple zeta valence
and quadruple zeta valence quality for H to Rn: Design and assessment
of accuracy. Phys. Chem. Chem. Phys..

[78] Blinc R. (1960). On the isotopic
effects in the ferroelectric behaviour of crystals with short hydrogen
bonds. J. Phys. Chem. Solids.

[79] McMahon M. I., Nelmes R. J., Kuhst W. F., Dorwarth R., Piltz R. O., Tun Z. (1990). Geometric effects of
deuteration on hydrogen-ordering phase transitions. Nature.

[80] Robertson J. M., Ubbelohde A. R. (1937). Isotope Effect in Hydrogen and Hydroxyl Bonds. Nature.

[81] Shibl M. F., Pietrzak M., Limbach H., Kühn O. (2007). Geometric
H/D Isotope Effects and Cooperativity of the Hydrogen Bonds in Porphycene. ChemPhysChem.

[82] Smith L. E., Briega-Martos V., Yang Y., Hammes-Schiffer S. (2025). Isotope Effects
for Water at Pt(111) Computed with Nuclear–Electronic Orbital
Theory. J. Phys. Chem. Lett..

[83] Åstrand P.-O., Karlström G., Engdahl A., Nelander B. (1995). Novel model for calculating
the intermolecular part of the infrared spectrum for molecular complexes. J. Chem. Phys..

[84] Swalina C., Wang Q., Chakraborty A., Hammes-Schiffer S. (2007). Analysis of
Nuclear Quantum Effects on Hydrogen Bonding. J. Phys. Chem. A.

[85] Ceriotti M., Fang W., Kusalik P. G., McKenzie R. H., Michaelides A., Morales M. A., Markland T. E. (2016). Nuclear Quantum Effects in Water
and Aqueous Systems: Experiment, Theory, and Current Challenges. Chem. Rev..

[86] Barone V. (2005). Anharmonic
vibrational properties by a fully automated second-order perturbative
approach. J. Chem. Phys..

[87] Tachikawa M., Mori K., Osamura Y. (1999). Isotope effect of hydrated clusters
of hydrogen chloride, HCl­(H_2_O)_
*n*
_ and DCl­(H_2_O)_
*n*
_ (*n* = 0–4): application of dynamic extended molecular orbital
method. Mol. Phys..

[88] Tachikawa M. (2002). Multi-component
molecular orbital theory for electrons and nuclei including many-body
effect with full configuration interaction treatment: isotope effects
on hydrogen molecules. Chem. Phys. Lett..

[89] Udagawa T., Ishimoto T., Tokiwa H., Tachikawa M., Nagashima U. (2004). The geometrical isotope effect of
C–H···O
type hydrogen bonds revealed by multi-component molecular orbital
calculation. Chem. Phys. Lett..

[90] Reyes A., Pak M. V., Hammes-Schiffer S. (2005). Investigation
of isotope effects
with the nuclear-electronic orbital approach. J. Chem. Phys..

[91] Udagawa T., Tachikawa M. (2006). H/D isotope
effect on porphine and porphycene molecules
with multicomponent hybrid density functional theory. J. Chem. Phys..

[92] González S. A., Aguirre N. F., Reyes A. (2008). Theoretical investigation of isotope
effects: The any-particle molecular orbital code. Int. J. Quantum Chem..

[93] Xu X., Yang Y. (2020). Full-quantum descriptions of molecular systems from constrained nuclear–electronic
orbital density functional theory. J. Chem.
Phys..

[94] Almlöf J. (1972). Hydrogen bond
studies. 71. Ab initio calculation of the vibrational structure and
equilibrium geometry in HF-2 and DF-2. Chem.
Phys. Lett..

[95] Tachikawa M., Shiga M. (2005). Geometrical H/D Isotope Effect on Hydrogen Bonds in Charged Water
Clusters. J. Am. Chem. Soc..

[96] Srinivasan V., Sebastiani D. (2011). The Isotope-Effect
in the Phase Transition of KH_2_PO_4_: New Insights
from Ab Initio Path-Integral
Simulations. J. Phys. Chem. C.

[97] Xue Y., Sexton T. M., Yang J., Tschumper G. S. (2024). Systematic
analysis of electronic barrier heights and widths for concerted proton
transfer in cyclic hydrogen bonded clusters: (HF)_
*n*
_ , (HCl)_
*n*
_ and (H_2_ O)_
*n*
_ where *n* = 3, 4, 5. Phys. Chem. Chem. Phys..

