# Supplementary material for: Nuclear–Electronic Orbital Coupled Cluster Theory with the Nuclear Hartree Product Representation for Multiple Quantum Nuclei and Application to Geometric Isotope Effects
Source: J Chem Theory Comput. 2026 Oct 7;22(20):10757–69. doi: 10.1021/acs.jctc.6c01568 (PMC13718558; doi:10.1021/acs.jctc.6c01568)
Supplement: Supplementary file 1 [file ct6c01568_si_001.pdf]

Supporting Information
Nuclear–Electronic Orbital Coupled Cluster Theory with the Nuclear Hartree Product Representation for Multiple Quantum Nuclei and Application to Geometric Isotope Effects

Rowan J. Goudy and Sharon Hammes-Schiffer*

Department of Chemistry, Princeton University, Princeton, NJ 08544, USA

E-mail: shs566@princeton.edu

Contents

S1 Alternative formulation of NEO-HF including self-Coulomb and self-exchange **S3**

S2 Additional comparisons of iterative convergence for NEO-CCSD t-amplitude equations **S4**

S3 Analysis of nuclear HP and SD occupied and virtual orbitals **S5**

S4 Perturbative triples correction is improved by HP when single excitations are excluded **S7**

S5 Nuclear HP NEO-CCSD t-amplitude programmable equations **S8**

S5.1 Nuclear HP NEO-CCSD correlation energy equation S8

S5.2 Nuclear HP NEO-CCSD t^{e}-amplitude equation S9

S5.3 Nuclear HP NEO-CCSD t^{ee}-amplitude equation S10

S5.4 Nuclear HP NEO-CCSD t^{n}-amplitude equation S11

S5.5 Nuclear HP NEO-CCSD t^{en}-amplitude equation S12

S6 Procedure for generating programmable equations when including double nuclear excitations **S13**

References **S15**

S1 Alternative formulation of NEO-HF including self-Coulomb and self-exchange

There is also another way to formulate the Fock operator in the nuclear HP representation that is analogous to the SD formulation for multiple quantum nuclei. Because we only care about $\varepsilon^{\mathrm{n}}_{\gamma,1}$ and not all $\varepsilon^{\mathrm{n}}_{\gamma,P}$, we can first note that

$$\left[J^{\mathrm{nn}}_{\gamma,\gamma}(\mathbf{x}^{\mathrm{n}}_{\gamma}) - K^{\mathrm{n}}_{\gamma}(\mathbf{x}^{\mathrm{n}}_{\gamma})\right]\chi^{\mathrm{n}}_{\gamma,1}(\mathbf{x}^{\mathrm{n}}_{\gamma}) = 0 \tag{S1}$$

where K^{n}_{γ} is the exchange operator for nucleus γ associated with $\chi^{\mathrm{n}}_{\gamma,1}$ and charge product $Z_{\gamma}Z_{\gamma}$. Therefore,

$$\left[F^{\mathrm{n}}_{\gamma}(\mathbf{x}^{\mathrm{n}}_{\gamma}) + J^{\mathrm{nn}}_{\gamma,\gamma}(\mathbf{x}^{\mathrm{n}}_{\gamma}) - K^{\mathrm{n}}_{\gamma}(\mathbf{x}^{\mathrm{n}}_{\gamma})\right]\chi^{\mathrm{n}}_{\gamma,1}(\mathbf{x}^{\mathrm{n}}_{\gamma}) = (\varepsilon^{\mathrm{n}}_{\gamma,1} + 0)\chi^{\mathrm{n}}_{\gamma,1}(\mathbf{x}^{\mathrm{n}}_{\gamma}) \tag{S2}$$

Thus, we can alternatively define a nuclear Fock operator and corresponding eigenvalue equation as

$$F'^{\mathrm{n}}_{\gamma}(\mathbf{x}^{\mathrm{n}}_{\gamma}) = F^{\mathrm{n}}_{\gamma}(\mathbf{x}^{\mathrm{n}}_{\gamma}) + J^{\mathrm{nn}}_{\gamma,\gamma}(\mathbf{x}^{\mathrm{n}}_{\gamma}) - K^{\mathrm{n}}_{\gamma}(\mathbf{x}^{\mathrm{n}}_{\gamma}) \tag{S3}$$

$$F'^{\mathrm{n}}_{\gamma}(\mathbf{x}^{\mathrm{n}}_{\gamma})\chi'^{\mathrm{n}}_{\gamma,P}(\mathbf{x}^{\mathrm{n}}_{\gamma}) = \varepsilon'^{\mathrm{n}}_{\gamma,P}\chi'^{\mathrm{n}}_{\gamma,P}(\mathbf{x}^{\mathrm{n}}_{\gamma}) \tag{S4}$$

where $\varepsilon'^{\mathrm{n}}_{\gamma,P}$ are not all the same as $\varepsilon^{\mathrm{n}}_{\gamma,P}$ from Eq. (4d). However, we have guaranteed that $\varepsilon'^{\mathrm{n}}_{\gamma,1} = \varepsilon^{\mathrm{n}}_{\gamma,1}$, and therefore the NEO-HF energy will be unchanged. NEO-HF performed with $F'^{\mathrm{n}}_{\gamma}(\mathbf{x}^{\mathrm{n}}_{\gamma})$ instead of $F^{\mathrm{n}}_{\gamma}(\mathbf{x}^{\mathrm{n}}_{\gamma})$ was found to have much slower convergence that was very similar to the nuclear SD representation. Thus, it is the inclusion of the $\left[J^{\mathrm{nn}}_{\gamma,\gamma}(\mathbf{x}^{\mathrm{n}}_{\gamma}) - K^{\mathrm{n}}_{\gamma}(\mathbf{x}^{\mathrm{n}}_{\gamma})\right]$ self-Coulomb and self-exchange terms, which are also implicitly included in the nuclear SD representation, that causes slow convergence.[1] Note that inclusion of self-Coulomb and self-exchange terms only impacts the virtual nuclear orbitals.

S2 Additional comparisons of iterative convergence for NEO-CCSD t-amplitude equations

The calculations in Table S1 are set up the same as those in Table 1 except that DIIS history is not truncated, and for the nuclear SD representation a DIIS extrapolation is performed after every iteration.

Table S1: Number of Iterations to Converge the NEO-CCSD t-Amplitude Equations when DIIS History is Not Truncated

System	SD	HP	Ratio
$H^+(H_2O)_4$ (Eigen)	68	11	6.2
$H^+(H_2O)_4$ (ring)	61	11	5.5
$H^+(H_2O)_4$ (*cis*-Zundel)	65	9	7.2
$H^+(H_2O)_4$ (*trans*-Zundel)	68	9	7.6
HCCH	47	11	4.3
H_2O	61	10	6.1
CH_3OH	59	13	4.5
CH_3NH_2	61	11	5.5
HCOOH	59	13	4.5
CH_2O	58	13	4.5
$(H_2O)_2$	61	11	5.5
$H^+(H_2O)_2$	51	9	5.7
Mean	60	11	5.6

The calculations in Table S2 are set up the same as those in Table 1 except that the convergence threshold is varied for calculations with DIIS, and the case without DIIS is tested with a loose convergence threshold. The nuclear HP representation provides a notable improvement in convergence speed at all thresholds tested and when DIIS is not used. The ratio for improvement does not depend significantly on the convergence threshold for the calculations with DIIS.

Table S2: Number of Iterations to Converge the NEO-CCSD t-amplitude Equations for H_2O with DIIS for Different Convergence Thresholds and without DIIS

DIIS	Threshold	SD	HP	Ratio
No	10^{-3}	257	6	42.8
Yes	10^{-3}	18	3	6.0
Yes	10^{-4}	27	5	5.4
Yes	10^{-5}	51	7	7.3
Yes	10^{-6}	72	11	6.5

S3 Analysis of nuclear HP and SD occupied and virtual orbitals

To visualize the differences between the nuclear HP virtual orbitals and the nuclear SD virtual orbitals, NEO-HF calculations were performed on HCCH with both protons treated quantum mechanically using the same basis sets as those used in Sections 4.1 and 4.2 from the main text. For one of the two quantum protons, the occupied orbital and the lowest-energy nondegenerate virtual orbital for each representation are shown in Figure S1. The radial probability density, $P(r)$, is calculated as $P(r) = 4\pi r^2 \langle \rho_{\mathrm{n}} \rangle$, where r is the distance from the basis function center and $\langle \rho_{\mathrm{n}} \rangle$ is the average nuclear density at that distance. The nuclear HP and SD representations produce nearly indistinguishable occupied orbital densities. The HP virtual orbital density resembles the density of the first excited state of a harmonic oscillator wavefunction, as expected for a stretching mode. The HP virtual orbital energy relative to the occupied orbital energy is 6300 cm^{-1}, around a factor of two greater than a typical stretching mode. However, the SD virtual orbital density is small along the bonding axis, and instead the virtual orbital density is predominantly located far away from the basis function center due to its Coulomb interaction with the occupied orbital density. The SD virtual orbital energy relative to the occupied orbital energy is 398,500 cm^{-1}, which is unphysically high and clearly not characteristic of a vibrational excitation.

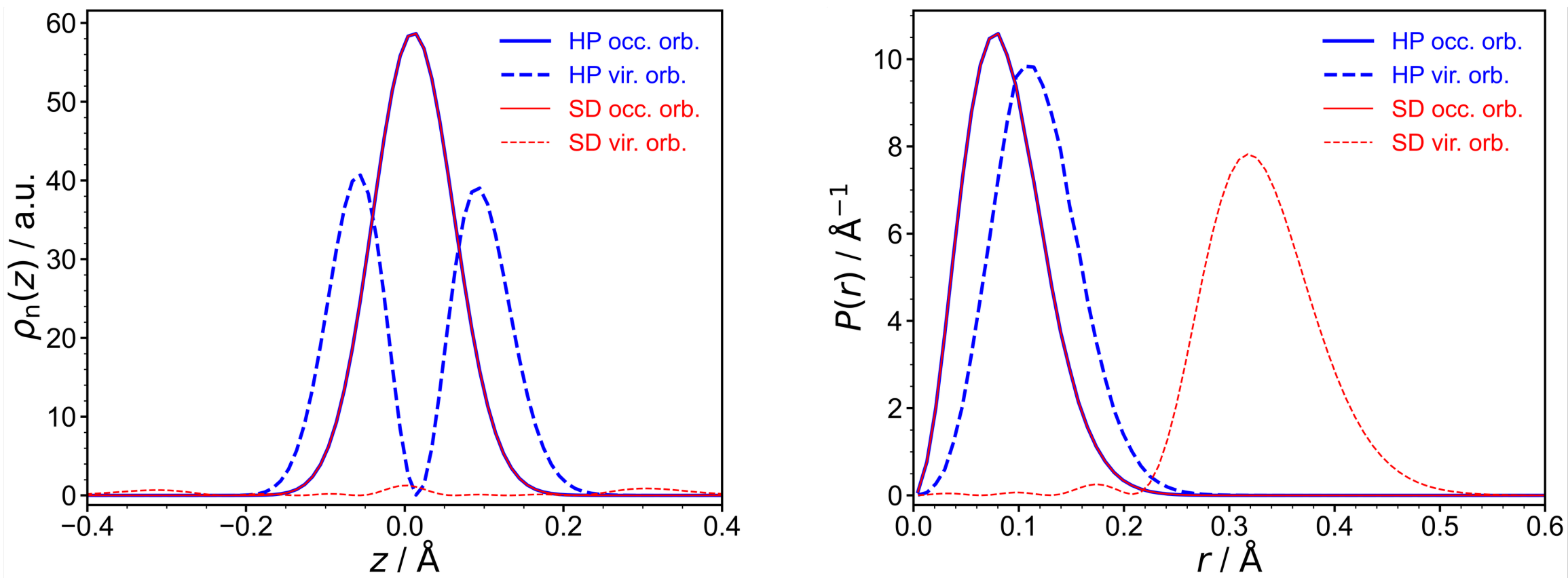

Figure S1: HCCH quantum proton orbital densities and probability distributions for occupied and virtual orbitals from a NEO-HF calculation with the proton basis function centers placed at −3.3216 Å and 0.0000 Å and the carbon nuclei placed at −2.2581 Å and −1.0635 Å along the z-axis. The orbital densities and probability distributions are plotted for the quantum proton with its basis function center at 0.0000 Å. The virtual orbitals shown correspond to the lowest-energy nondegenerate virtual orbital for each representation. (left) Nuclear orbital density along the z-axis. (right) Radial probability distribution from the basis function center. In both panels, the occupied orbitals are visually indistinguishable, and the virtual orbitals are qualitatively different for the nuclear HP and SD representations.

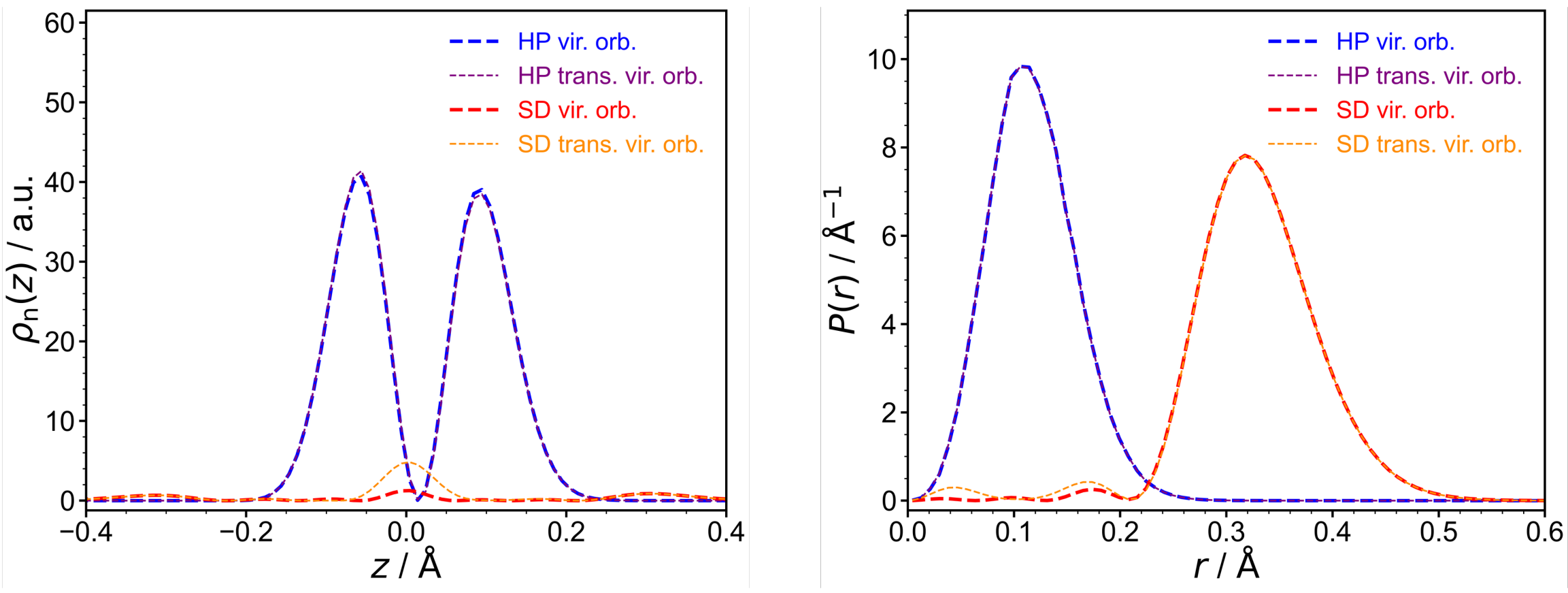

Figure S2: HCCH quantum proton orbital densities and probability distributions for virtual orbitals from NEO calculations with the proton basis function centers placed at −3.3216 Å and 0.0000 Å and the carbon nuclei placed at −2.2581 Å and −1.0635 Å along the z-axis. The orbital densities and probability distributions are plotted for the quantum proton with its basis function center at 0.0000 Å. The NEO-HF virtual orbitals shown in red and blue correspond to the lowest-energy nondegenerate virtual orbital for each representation. Also shown are the same virtual orbitals after transformation using the converged NEO-CCSD single nuclear amplitudes. For the nuclear SD representation, the transformed virtual MO coefficients, $X^{\mathrm{n}}_{\mu A}$, were calculated as $X^{\mathrm{n}}_{\mu A} = C^{\mathrm{n}}_{\mu A} - \sum_I C^{\mathrm{n}}_{\mu I} t^I_A$, where μ are the atomic orbitals, I are the occupied nuclear orbitals, and A are the virtual nuclear orbitals.[2] For the nuclear HP representation, the transformed virtual MO coefficients for nucleus γ, $(X^{\mathrm{n}}_{\gamma})_{\mu A}$, were calculated as $(X^{\mathrm{n}}_{\gamma})_{\mu A} = (C^{\mathrm{n}}_{\gamma})_{\mu A} - \sum_{I \in \mathcal{S}^{\mathrm{n(o)}}_{\gamma}} (C^{\mathrm{n}}_{\gamma})_{\mu I} (t^{\mathrm{n}}_{\gamma})^I_A$, where μ are the atomic orbitals for γ and $A \in \mathcal{S}^{\mathrm{n(v)}}_{\gamma}$. (left) Nuclear orbital density along the z-axis. (right) Radial probability distribution from the basis function center. In both panels, the nuclear SD and nuclear HP virtual orbitals remain qualitatively different from each other after transformation.

S4 Perturbative triples correction is improved by HP when single excitations are excluded

The calculations in Table S3 use the same setup as those in Table 2 except that single excitations are excluded. The nuclear HP representation provides a notable improvement in

the perturbative triples correction even when single excitations are excluded.

Table S3: Percent Errors in Correlation Energy for CH_3OH Compared to NEO-CCDT$_{\text{een}}$ SD

Method	Corr. error
NEO-CCD	0.75%
NEO-CCD(T$_{\text{een}}$) SD	0.38%
NEO-CCD(T$_{\text{een}}$) HP	0.09%

S5 Nuclear HP NEO-CCSD t-amplitude programmable equations

Programmable spin orbital equations for implementing NEO-CCSD within the nuclear HP representation are provided below. Einstein notation where repeated orbital indices are implicitly summed over is used here. Orbital indices used are $i, j, k, l \in \mathcal{S}^{\text{e(o)}}$ and $a, b, c, d \in \mathcal{S}^{\text{e(v)}}$. Subsections S5.1–S5.3 also use $I, J, K, L \in \mathcal{S}_\gamma^{\text{n(o)}}$, $A, B, C, D \in \mathcal{S}_\gamma^{\text{n(v)}}$, $M, N, O, P \in \mathcal{S}_\eta^{\text{n(o)}}$, and $E, F, G, H \in \mathcal{S}_\eta^{\text{n(v)}}$. Subsections S5.4–S5.5 instead use $I, J, K, L \in \mathcal{S}_\kappa^{\text{n(o)}}$, $A, B, C, D \in \mathcal{S}_\kappa^{\text{n(v)}}$, $M, N, O, P \in \mathcal{S}_\xi^{\text{n(o)}}$, and $E, F, G, H \in \mathcal{S}_\xi^{\text{n(v)}}$. See also Eqs. (11a)–(11e) from the main text.

S5.1 Nuclear HP NEO-CCSD correlation energy equation

$$E_{\text{NEO}} = \langle 0^{\text{e}}0^{\text{n}}|\bar{H}_{\text{NEO}}|0^{\text{e}}0^{\text{n}}\rangle$$

$$E_{\text{NEO}} = (F^{\text{e}})_i^a(t^{\text{e}})_a^i + \tfrac{1}{4}(\bar{g}^{\text{ee}})_{ij}^{ab}(t^{\text{ee}})_{ba}^{ji} - \tfrac{1}{2}(\bar{g}^{\text{ee}})_{ij}^{ab}(t^{\text{e}})_b^i(t^{\text{e}})_a^j + \textstyle\sum_{\gamma\in\mathcal{N}_{\text{q}}}\big[(F_\gamma^{\text{n}})_I^A(t_\gamma^{\text{n}})_A^I + (g_\gamma^{\text{en}})_{iI}^{aA}(t_\gamma^{\text{en}})_{aA}^{iI} + (g_\gamma^{\text{en}})_{iI}^{aA}(t^{\text{e}})_a^i(t_\gamma^{\text{n}})_A^I\big] + \textstyle\sum_{\substack{\gamma,\eta\in\mathcal{N}_{\text{q}}\\ \gamma<\eta}}\big[(g_{\gamma\eta}^{\text{nn}})_{IM}^{AE}(t_\gamma^{\text{n}})_A^I(t_\eta^{\text{n}})_E^M\big] \tag{S5}$$

S5.2 Nuclear HP NEO-CCSD t^{e}-amplitude equation

$$0 = \langle \mu^{\mathrm{e}} | \bar{H}_{\mathrm{NEO}} | 0^{\mathrm{e}} 0^{\mathrm{n}} \rangle$$

$$0 = (F^{\mathrm{e}})^{i}_{a} - (F^{\mathrm{e}})^{i}_{j}(t^{\mathrm{e}})^{j}_{a} + (F^{\mathrm{e}})^{b}_{a}(t^{\mathrm{e}})^{i}_{b} - (F^{\mathrm{e}})^{b}_{j}(t^{\mathrm{ee}})^{ji}_{ab} - (\bar{g}^{\mathrm{ee}})^{ib}_{ja}(t^{\mathrm{e}})^{j}_{b} + \tfrac{1}{2}(\bar{g}^{\mathrm{ee}})^{ib}_{jk}(t^{\mathrm{ee}})^{kj}_{ab} - \tfrac{1}{2}(\bar{g}^{\mathrm{ee}})^{bc}_{ja}(t^{\mathrm{ee}})^{ji}_{cb} - (F^{\mathrm{e}})^{b}_{j}(t^{\mathrm{e}})^{i}_{b}(t^{\mathrm{e}})^{j}_{a} - (\bar{g}^{\mathrm{ee}})^{ib}_{jk}(t^{\mathrm{e}})^{j}_{a}(t^{\mathrm{e}})^{k}_{b} + (\bar{g}^{\mathrm{ee}})^{bc}_{ja}(t^{\mathrm{e}})^{i}_{c}(t^{\mathrm{e}})^{j}_{b} - \tfrac{1}{2}(\bar{g}^{\mathrm{ee}})^{bc}_{jk}(t^{\mathrm{e}})^{j}_{a}(t^{\mathrm{ee}})^{ki}_{cb} - \tfrac{1}{2}(\bar{g}^{\mathrm{ee}})^{bc}_{jk}(t^{\mathrm{e}})^{i}_{c}(t^{\mathrm{ee}})^{kj}_{ab} + (\bar{g}^{\mathrm{ee}})^{bc}_{jk}(t^{\mathrm{e}})^{j}_{c}(t^{\mathrm{ee}})^{ki}_{ab} + (\bar{g}^{\mathrm{ee}})^{bc}_{jk}(t^{\mathrm{e}})^{i}_{c}(t^{\mathrm{e}})^{j}_{a}(t^{\mathrm{e}})^{k}_{b} + \sum_{\gamma\in\mathcal{N}_{\mathrm{q}}}\Big[(F^{\mathrm{n}}_{\gamma})^{A}_{I}(t^{\mathrm{en}}_{\gamma})^{iI}_{aA} + (g^{\mathrm{en}}_{\gamma})^{iA}_{aI}(t^{\mathrm{n}}_{\gamma})^{I}_{A} - (g^{\mathrm{en}}_{\gamma})^{iA}_{jI}(t^{\mathrm{en}}_{\gamma})^{jI}_{aA} + (g^{\mathrm{en}}_{\gamma})^{bA}_{aI}(t^{\mathrm{en}}_{\gamma})^{iI}_{bA} - (g^{\mathrm{en}}_{\gamma})^{iA}_{jI}(t^{\mathrm{e}})^{j}_{a}(t^{\mathrm{n}}_{\gamma})^{I}_{A} + (g^{\mathrm{en}}_{\gamma})^{bA}_{aI}(t^{\mathrm{e}})^{i}_{b}(t^{\mathrm{n}}_{\gamma})^{I}_{A} - (g^{\mathrm{en}}_{\gamma})^{bA}_{jI}(t^{\mathrm{e}})^{j}_{a}(t^{\mathrm{en}}_{\gamma})^{iI}_{bA} - (g^{\mathrm{en}}_{\gamma})^{bA}_{jI}(t^{\mathrm{e}})^{i}_{b}(t^{\mathrm{en}}_{\gamma})^{jI}_{aA} + (g^{\mathrm{en}}_{\gamma})^{bA}_{jI}(t^{\mathrm{e}})^{j}_{b}(t^{\mathrm{en}}_{\gamma})^{iI}_{aA} - (g^{\mathrm{en}}_{\gamma})^{bA}_{jI}(t^{\mathrm{n}}_{\gamma})^{I}_{A}(t^{\mathrm{ee}})^{ji}_{ab} - (g^{\mathrm{en}}_{\gamma})^{bA}_{jI}(t^{\mathrm{e}})^{i}_{b}(t^{\mathrm{e}})^{j}_{a}(t^{\mathrm{n}}_{\gamma})^{I}_{A}\Big] + \sum_{\substack{\gamma,\eta\in\mathcal{N}_{\mathrm{q}}\\ \gamma<\eta}}\Big[(g^{\mathrm{nn}}_{\gamma\eta})^{AE}_{IM}(t^{\mathrm{n}}_{\gamma})^{I}_{A}(t^{\mathrm{en}}_{\eta})^{iM}_{aE} + (g^{\mathrm{nn}}_{\gamma\eta})^{AE}_{IM}(t^{\mathrm{n}}_{\eta})^{M}_{E}(t^{\mathrm{en}}_{\gamma})^{iI}_{aA}\Big] \tag{S6}$$

S5.3 Nuclear HP NEO-CCSD t^{ee}-amplitude equation

$$0 = \langle \mu^{\mathrm{ee}} | \bar{H}_{\mathrm{NEO}} | 0^{\mathrm{e}} 0^{\mathrm{n}} \rangle$$

$$\begin{aligned}
0 = {} & (\bar{g}^{\mathrm{ee}})^{ji}_{ba} + (F^{\mathrm{e}})^{i}_{k}(t^{\mathrm{ee}})^{kj}_{ba} - (F^{\mathrm{e}})^{j}_{k}(t^{\mathrm{ee}})^{ki}_{ba} + (F^{\mathrm{e}})^{c}_{a}(t^{\mathrm{ee}})^{ji}_{bc} - (F^{\mathrm{e}})^{c}_{b}(t^{\mathrm{ee}})^{ji}_{ac} - (\bar{g}^{\mathrm{ee}})^{ji}_{ka}(t^{\mathrm{e}})^{k}_{b} + (\bar{g}^{\mathrm{ee}})^{ji}_{kb}(t^{\mathrm{e}})^{k}_{a} - \\
& (\bar{g}^{\mathrm{ee}})^{ic}_{ba}(t^{\mathrm{e}})^{j}_{c} + (\bar{g}^{\mathrm{ee}})^{jc}_{ba}(t^{\mathrm{e}})^{i}_{c} - \tfrac{1}{2}(\bar{g}^{\mathrm{ee}})^{ji}_{kl}(t^{\mathrm{ee}})^{lk}_{ba} + (\bar{g}^{\mathrm{ee}})^{ic}_{ka}(t^{\mathrm{ee}})^{kj}_{bc} - (\bar{g}^{\mathrm{ee}})^{jc}_{ka}(t^{\mathrm{ee}})^{ki}_{bc} - (\bar{g}^{\mathrm{ee}})^{ic}_{kb}(t^{\mathrm{ee}})^{kj}_{ac} + \\
& (\bar{g}^{\mathrm{ee}})^{jc}_{kb}(t^{\mathrm{ee}})^{ki}_{ac} - \tfrac{1}{2}(\bar{g}^{\mathrm{ee}})^{cd}_{ba}(t^{\mathrm{ee}})^{ji}_{dc} - (F^{\mathrm{e}})^{c}_{k}(t^{\mathrm{e}})^{k}_{a}(t^{\mathrm{ee}})^{ji}_{bc} + (F^{\mathrm{e}})^{c}_{k}(t^{\mathrm{e}})^{k}_{b}(t^{\mathrm{ee}})^{ji}_{ac} + (F^{\mathrm{e}})^{c}_{k}(t^{\mathrm{e}})^{i}_{c}(t^{\mathrm{ee}})^{kj}_{ba} - \\
& (F^{\mathrm{e}})^{c}_{k}(t^{\mathrm{e}})^{j}_{c}(t^{\mathrm{ee}})^{ki}_{ba} + (\bar{g}^{\mathrm{ee}})^{ji}_{kl}(t^{\mathrm{e}})^{k}_{b}(t^{\mathrm{e}})^{l}_{a} + (\bar{g}^{\mathrm{ee}})^{ic}_{ka}(t^{\mathrm{e}})^{j}_{c}(t^{\mathrm{e}})^{k}_{b} - (\bar{g}^{\mathrm{ee}})^{jc}_{ka}(t^{\mathrm{e}})^{i}_{c}(t^{\mathrm{e}})^{k}_{b} - (\bar{g}^{\mathrm{ee}})^{ic}_{kb}(t^{\mathrm{e}})^{j}_{c}(t^{\mathrm{e}})^{k}_{a} + \\
& (\bar{g}^{\mathrm{ee}})^{jc}_{kb}(t^{\mathrm{e}})^{i}_{c}(t^{\mathrm{e}})^{k}_{a} + (\bar{g}^{\mathrm{ee}})^{cd}_{ba}(t^{\mathrm{e}})^{i}_{d}(t^{\mathrm{e}})^{j}_{c} + (\bar{g}^{\mathrm{ee}})^{ic}_{kl}(t^{\mathrm{e}})^{k}_{a}(t^{\mathrm{ee}})^{lj}_{bc} - (\bar{g}^{\mathrm{ee}})^{ic}_{kl}(t^{\mathrm{e}})^{k}_{b}(t^{\mathrm{ee}})^{lj}_{ac} + \tfrac{1}{2}(\bar{g}^{\mathrm{ee}})^{ic}_{kl}(t^{\mathrm{e}})^{j}_{c}(t^{\mathrm{ee}})^{lk}_{ba} - \\
& (\bar{g}^{\mathrm{ee}})^{ic}_{kl}(t^{\mathrm{e}})^{k}_{c}(t^{\mathrm{ee}})^{lj}_{ba} - (\bar{g}^{\mathrm{ee}})^{jc}_{kl}(t^{\mathrm{e}})^{k}_{a}(t^{\mathrm{ee}})^{li}_{bc} + (\bar{g}^{\mathrm{ee}})^{jc}_{kl}(t^{\mathrm{e}})^{k}_{b}(t^{\mathrm{ee}})^{li}_{ac} - \tfrac{1}{2}(\bar{g}^{\mathrm{ee}})^{jc}_{kl}(t^{\mathrm{e}})^{i}_{c}(t^{\mathrm{ee}})^{lk}_{ba} + (\bar{g}^{\mathrm{ee}})^{jc}_{kl}(t^{\mathrm{e}})^{k}_{c}(t^{\mathrm{ee}})^{li}_{ba} + \\
& \tfrac{1}{2}(\bar{g}^{\mathrm{ee}})^{cd}_{ka}(t^{\mathrm{e}})^{k}_{b}(t^{\mathrm{ee}})^{ji}_{dc} - (\bar{g}^{\mathrm{ee}})^{cd}_{ka}(t^{\mathrm{e}})^{i}_{d}(t^{\mathrm{ee}})^{kj}_{bc} + (\bar{g}^{\mathrm{ee}})^{cd}_{ka}(t^{\mathrm{e}})^{j}_{d}(t^{\mathrm{ee}})^{ki}_{bc} - (\bar{g}^{\mathrm{ee}})^{cd}_{ka}(t^{\mathrm{e}})^{k}_{d}(t^{\mathrm{ee}})^{ji}_{bc} - \\
& \tfrac{1}{2}(\bar{g}^{\mathrm{ee}})^{cd}_{kb}(t^{\mathrm{e}})^{k}_{a}(t^{\mathrm{ee}})^{ji}_{dc} + (\bar{g}^{\mathrm{ee}})^{cd}_{kb}(t^{\mathrm{e}})^{i}_{d}(t^{\mathrm{ee}})^{kj}_{ac} - (\bar{g}^{\mathrm{ee}})^{cd}_{kb}(t^{\mathrm{e}})^{j}_{d}(t^{\mathrm{ee}})^{ki}_{ac} + (\bar{g}^{\mathrm{ee}})^{cd}_{kb}(t^{\mathrm{e}})^{k}_{d}(t^{\mathrm{ee}})^{ji}_{ac} + \\
& \tfrac{1}{2}(\bar{g}^{\mathrm{ee}})^{cd}_{kl}(t^{\mathrm{ee}})^{ji}_{ad}(t^{\mathrm{ee}})^{lk}_{bc} - (\bar{g}^{\mathrm{ee}})^{cd}_{kl}(t^{\mathrm{ee}})^{ki}_{ad}(t^{\mathrm{ee}})^{lj}_{bc} - \tfrac{1}{2}(\bar{g}^{\mathrm{ee}})^{cd}_{kl}(t^{\mathrm{ee}})^{ki}_{ba}(t^{\mathrm{ee}})^{lj}_{dc} - \tfrac{1}{2}(\bar{g}^{\mathrm{ee}})^{cd}_{kl}(t^{\mathrm{ee}})^{ji}_{bd}(t^{\mathrm{ee}})^{lk}_{ac} + \\
& (\bar{g}^{\mathrm{ee}})^{cd}_{kl}(t^{\mathrm{ee}})^{ki}_{bd}(t^{\mathrm{ee}})^{lj}_{ac} + \tfrac{1}{4}(\bar{g}^{\mathrm{ee}})^{cd}_{kl}(t^{\mathrm{ee}})^{ji}_{dc}(t^{\mathrm{ee}})^{lk}_{ba} - \tfrac{1}{2}(\bar{g}^{\mathrm{ee}})^{cd}_{kl}(t^{\mathrm{ee}})^{ki}_{dc}(t^{\mathrm{ee}})^{lj}_{ba} - (\bar{g}^{\mathrm{ee}})^{ic}_{kl}(t^{\mathrm{e}})^{j}_{c}(t^{\mathrm{e}})^{k}_{b}(t^{\mathrm{e}})^{l}_{a} + \\
& (\bar{g}^{\mathrm{ee}})^{jc}_{kl}(t^{\mathrm{e}})^{i}_{c}(t^{\mathrm{e}})^{k}_{b}(t^{\mathrm{e}})^{l}_{a} - (\bar{g}^{\mathrm{ee}})^{cd}_{ka}(t^{\mathrm{e}})^{i}_{d}(t^{\mathrm{e}})^{j}_{c}(t^{\mathrm{e}})^{k}_{b} + (\bar{g}^{\mathrm{ee}})^{cd}_{kb}(t^{\mathrm{e}})^{i}_{d}(t^{\mathrm{e}})^{j}_{c}(t^{\mathrm{e}})^{k}_{a} - (\bar{g}^{\mathrm{ee}})^{cd}_{kl}(t^{\mathrm{e}})^{k}_{a}(t^{\mathrm{e}})^{l}_{d}(t^{\mathrm{ee}})^{ji}_{bc} - \\
& \tfrac{1}{2}(\bar{g}^{\mathrm{ee}})^{cd}_{kl}(t^{\mathrm{e}})^{k}_{b}(t^{\mathrm{e}})^{l}_{a}(t^{\mathrm{ee}})^{ji}_{dc} + (\bar{g}^{\mathrm{ee}})^{cd}_{kl}(t^{\mathrm{e}})^{k}_{b}(t^{\mathrm{e}})^{l}_{d}(t^{\mathrm{ee}})^{ji}_{ac} - (\bar{g}^{\mathrm{ee}})^{cd}_{kl}(t^{\mathrm{e}})^{i}_{d}(t^{\mathrm{e}})^{k}_{a}(t^{\mathrm{ee}})^{lj}_{bc} + (\bar{g}^{\mathrm{ee}})^{cd}_{kl}(t^{\mathrm{e}})^{i}_{d}(t^{\mathrm{e}})^{k}_{b}(t^{\mathrm{ee}})^{lj}_{ac} - \\
& \tfrac{1}{2}(\bar{g}^{\mathrm{ee}})^{cd}_{kl}(t^{\mathrm{e}})^{i}_{d}(t^{\mathrm{e}})^{j}_{c}(t^{\mathrm{ee}})^{lk}_{ba} + (\bar{g}^{\mathrm{ee}})^{cd}_{kl}(t^{\mathrm{e}})^{i}_{d}(t^{\mathrm{e}})^{k}_{c}(t^{\mathrm{ee}})^{lj}_{ba} + (\bar{g}^{\mathrm{ee}})^{cd}_{kl}(t^{\mathrm{e}})^{j}_{d}(t^{\mathrm{e}})^{k}_{a}(t^{\mathrm{ee}})^{li}_{bc} - (\bar{g}^{\mathrm{ee}})^{cd}_{kl}(t^{\mathrm{e}})^{j}_{d}(t^{\mathrm{e}})^{k}_{b}(t^{\mathrm{ee}})^{li}_{ac} - \\
& (\bar{g}^{\mathrm{ee}})^{cd}_{kl}(t^{\mathrm{e}})^{j}_{d}(t^{\mathrm{e}})^{k}_{c}(t^{\mathrm{ee}})^{li}_{ba} + (\bar{g}^{\mathrm{ee}})^{cd}_{kl}(t^{\mathrm{e}})^{i}_{d}(t^{\mathrm{e}})^{j}_{c}(t^{\mathrm{e}})^{k}_{b}(t^{\mathrm{e}})^{l}_{a} + \textstyle\sum_{\gamma \in \mathcal{N}_{\mathrm{q}}} \big[(g^{\mathrm{en}}_{\gamma})^{iA}_{aI}(t^{\mathrm{en}}_{\gamma})^{jI}_{bA} - (g^{\mathrm{en}}_{\gamma})^{jA}_{aI}(t^{\mathrm{en}}_{\gamma})^{iI}_{bA} - \\
& (g^{\mathrm{en}}_{\gamma})^{iA}_{bI}(t^{\mathrm{en}}_{\gamma})^{jI}_{aA} + (g^{\mathrm{en}}_{\gamma})^{jA}_{bI}(t^{\mathrm{en}}_{\gamma})^{iI}_{aA} - (g^{\mathrm{en}}_{\gamma})^{iA}_{kI}(t^{\mathrm{e}})^{k}_{a}(t^{\mathrm{en}}_{\gamma})^{jI}_{bA} + (g^{\mathrm{en}}_{\gamma})^{iA}_{kI}(t^{\mathrm{e}})^{k}_{b}(t^{\mathrm{en}}_{\gamma})^{jI}_{aA} + (g^{\mathrm{en}}_{\gamma})^{iA}_{kI}(t^{\mathrm{n}}_{\gamma})^{I}_{A}(t^{\mathrm{ee}})^{kj}_{ba} + \\
& (g^{\mathrm{en}}_{\gamma})^{jA}_{kI}(t^{\mathrm{e}})^{k}_{a}(t^{\mathrm{en}}_{\gamma})^{iI}_{bA} - (g^{\mathrm{en}}_{\gamma})^{jA}_{kI}(t^{\mathrm{e}})^{k}_{b}(t^{\mathrm{en}}_{\gamma})^{iI}_{aA} - (g^{\mathrm{en}}_{\gamma})^{jA}_{kI}(t^{\mathrm{n}}_{\gamma})^{I}_{A}(t^{\mathrm{ee}})^{ki}_{ba} + (g^{\mathrm{en}}_{\gamma})^{cA}_{aI}(t^{\mathrm{e}})^{i}_{c}(t^{\mathrm{en}}_{\gamma})^{jI}_{bA} - \\
& (g^{\mathrm{en}}_{\gamma})^{cA}_{aI}(t^{\mathrm{e}})^{j}_{c}(t^{\mathrm{en}}_{\gamma})^{iI}_{bA} + (g^{\mathrm{en}}_{\gamma})^{cA}_{aI}(t^{\mathrm{n}}_{\gamma})^{I}_{A}(t^{\mathrm{ee}})^{ji}_{bc} - (g^{\mathrm{en}}_{\gamma})^{cA}_{bI}(t^{\mathrm{e}})^{i}_{c}(t^{\mathrm{en}}_{\gamma})^{jI}_{aA} + (g^{\mathrm{en}}_{\gamma})^{cA}_{bI}(t^{\mathrm{e}})^{j}_{c}(t^{\mathrm{en}}_{\gamma})^{iI}_{aA} - \\
& (g^{\mathrm{en}}_{\gamma})^{cA}_{bI}(t^{\mathrm{n}}_{\gamma})^{I}_{A}(t^{\mathrm{ee}})^{ji}_{ac} + (g^{\mathrm{en}}_{\gamma})^{cA}_{kI}(t^{\mathrm{ee}})^{ji}_{ac}(t^{\mathrm{en}}_{\gamma})^{kI}_{bA} - (g^{\mathrm{en}}_{\gamma})^{cA}_{kI}(t^{\mathrm{ee}})^{ki}_{ac}(t^{\mathrm{en}}_{\gamma})^{jI}_{bA} + (g^{\mathrm{en}}_{\gamma})^{cA}_{kI}(t^{\mathrm{ee}})^{kj}_{ac}(t^{\mathrm{en}}_{\gamma})^{iI}_{bA} - \\
& (g^{\mathrm{en}}_{\gamma})^{cA}_{kI}(t^{\mathrm{ee}})^{ki}_{ba}(t^{\mathrm{en}}_{\gamma})^{jI}_{cA} + (g^{\mathrm{en}}_{\gamma})^{cA}_{kI}(t^{\mathrm{ee}})^{kj}_{ba}(t^{\mathrm{en}}_{\gamma})^{iI}_{cA} - (g^{\mathrm{en}}_{\gamma})^{cA}_{kI}(t^{\mathrm{ee}})^{ji}_{bc}(t^{\mathrm{en}}_{\gamma})^{kI}_{aA} + (g^{\mathrm{en}}_{\gamma})^{cA}_{kI}(t^{\mathrm{ee}})^{ki}_{bc}(t^{\mathrm{en}}_{\gamma})^{jI}_{aA} - \\
& (g^{\mathrm{en}}_{\gamma})^{cA}_{kI}(t^{\mathrm{ee}})^{kj}_{bc}(t^{\mathrm{en}}_{\gamma})^{iI}_{aA} - (g^{\mathrm{en}}_{\gamma})^{cA}_{kI}(t^{\mathrm{e}})^{k}_{a}(t^{\mathrm{n}}_{\gamma})^{I}_{A}(t^{\mathrm{ee}})^{ji}_{bc} + (g^{\mathrm{en}}_{\gamma})^{cA}_{kI}(t^{\mathrm{e}})^{k}_{b}(t^{\mathrm{n}}_{\gamma})^{I}_{A}(t^{\mathrm{ee}})^{ji}_{ac} - (g^{\mathrm{en}}_{\gamma})^{cA}_{kI}(t^{\mathrm{e}})^{i}_{c}(t^{\mathrm{e}})^{k}_{a}(t^{\mathrm{en}}_{\gamma})^{jI}_{bA} + \\
& (g^{\mathrm{en}}_{\gamma})^{cA}_{kI}(t^{\mathrm{e}})^{i}_{c}(t^{\mathrm{e}})^{k}_{b}(t^{\mathrm{en}}_{\gamma})^{jI}_{aA} + (g^{\mathrm{en}}_{\gamma})^{cA}_{kI}(t^{\mathrm{e}})^{i}_{c}(t^{\mathrm{n}}_{\gamma})^{I}_{A}(t^{\mathrm{ee}})^{kj}_{ba} + (g^{\mathrm{en}}_{\gamma})^{cA}_{kI}(t^{\mathrm{e}})^{j}_{c}(t^{\mathrm{e}})^{k}_{a}(t^{\mathrm{en}}_{\gamma})^{iI}_{bA} - \\
& (g^{\mathrm{en}}_{\gamma})^{cA}_{kI}(t^{\mathrm{e}})^{j}_{c}(t^{\mathrm{e}})^{k}_{b}(t^{\mathrm{en}}_{\gamma})^{iI}_{aA} - (g^{\mathrm{en}}_{\gamma})^{cA}_{kI}(t^{\mathrm{e}})^{j}_{c}(t^{\mathrm{n}}_{\gamma})^{I}_{A}(t^{\mathrm{ee}})^{ki}_{ba} \big] + \textstyle\sum_{\substack{\gamma,\eta \in \mathcal{N}_{\mathrm{q}} \\ \gamma < \eta}} \big[(g^{\mathrm{nn}}_{\gamma\eta})^{AE}_{IM}(t^{\mathrm{en}}_{\gamma})^{iI}_{aA}(t^{\mathrm{en}}_{\eta})^{jM}_{bE} - \\
& (g^{\mathrm{nn}}_{\gamma\eta})^{AE}_{IM}(t^{\mathrm{en}}_{\gamma})^{jI}_{aA}(t^{\mathrm{en}}_{\eta})^{iM}_{bE} - (g^{\mathrm{nn}}_{\gamma\eta})^{AE}_{IM}(t^{\mathrm{en}}_{\gamma})^{iI}_{bA}(t^{\mathrm{en}}_{\eta})^{jM}_{aE} + (g^{\mathrm{nn}}_{\gamma\eta})^{AE}_{IM}(t^{\mathrm{en}}_{\gamma})^{jI}_{bA}(t^{\mathrm{en}}_{\eta})^{iM}_{aE} \big]
\end{aligned} \tag{S7}$$

S5.4 Nuclear HP NEO-CCSD t^{n}-amplitude equation

$$0 = \langle \mu^{\mathrm{n}}_{\kappa} | \bar{H}_{\mathrm{NEO}} | 0^{\mathrm{e}} 0^{\mathrm{n}} \rangle$$

$$0 = (F^{\mathrm{n}}_{\kappa})^{I}_{A} - (F^{\mathrm{n}}_{\kappa})^{I}_{J} (t^{\mathrm{n}}_{\kappa})^{J}_{A} + (F^{\mathrm{n}}_{\kappa})^{B}_{A} (t^{\mathrm{n}}_{\kappa})^{I}_{B} + (F^{\mathrm{e}})^{a}_{i} (t^{\mathrm{en}}_{\kappa})^{iI}_{aA} + (g^{\mathrm{en}}_{\kappa})^{aI}_{iA} (t^{\mathrm{e}})^{i}_{a} - (g^{\mathrm{en}}_{\kappa})^{aI}_{iJ} (t^{\mathrm{en}}_{\kappa})^{iJ}_{aA} + (g^{\mathrm{en}}_{\kappa})^{aB}_{iA} (t^{\mathrm{en}}_{\kappa})^{iI}_{aB} - (F^{\mathrm{n}}_{\kappa})^{B}_{J} (t^{\mathrm{n}}_{\kappa})^{I}_{B} (t^{\mathrm{n}}_{\kappa})^{J}_{A} - (g^{\mathrm{en}}_{\kappa})^{aI}_{iJ} (t^{\mathrm{e}})^{i}_{a} (t^{\mathrm{n}}_{\kappa})^{J}_{A} + (g^{\mathrm{en}}_{\kappa})^{aB}_{iA} (t^{\mathrm{e}})^{i}_{a} (t^{\mathrm{n}}_{\kappa})^{I}_{B} - (\bar{g}^{\mathrm{ee}})^{ab}_{ij} (t^{\mathrm{e}})^{i}_{b} (t^{\mathrm{en}}_{\kappa})^{jI}_{aA} - (g^{\mathrm{en}}_{\kappa})^{aB}_{iJ} (t^{\mathrm{n}}_{\kappa})^{J}_{A} (t^{\mathrm{en}}_{\kappa})^{iI}_{aB} - (g^{\mathrm{en}}_{\kappa})^{aB}_{iJ} (t^{\mathrm{n}}_{\kappa})^{I}_{B} (t^{\mathrm{en}}_{\kappa})^{iJ}_{aA} + (g^{\mathrm{en}}_{\kappa})^{aB}_{iJ} (t^{\mathrm{n}}_{\kappa})^{J}_{B} (t^{\mathrm{en}}_{\kappa})^{iI}_{aA} - (g^{\mathrm{en}}_{\kappa})^{aB}_{iJ} (t^{\mathrm{e}})^{i}_{a} (t^{\mathrm{n}}_{\kappa})^{I}_{B} (t^{\mathrm{n}}_{\kappa})^{J}_{A} + \sum_{\xi \in \mathcal{N}_{\mathrm{r}}} \Big[(g^{\mathrm{nn}}_{\kappa\xi})^{IE}_{AM} (t^{\mathrm{n}}_{\xi})^{M}_{E} - (g^{\mathrm{nn}}_{\kappa\xi})^{IE}_{JM} (t^{\mathrm{n}}_{\kappa})^{J}_{A} (t^{\mathrm{n}}_{\xi})^{M}_{E} + (g^{\mathrm{nn}}_{\kappa\xi})^{BE}_{AM} (t^{\mathrm{n}}_{\kappa})^{I}_{B} (t^{\mathrm{n}}_{\xi})^{M}_{E} + (g^{\mathrm{en}}_{\xi})^{aE}_{iM} (t^{\mathrm{n}}_{\xi})^{M}_{E} (t^{\mathrm{en}}_{\kappa})^{iI}_{aA} - (g^{\mathrm{nn}}_{\kappa\xi})^{BE}_{JM} (t^{\mathrm{n}}_{\kappa})^{I}_{B} (t^{\mathrm{n}}_{\kappa})^{J}_{A} (t^{\mathrm{n}}_{\xi})^{M}_{E} \Big] \tag{S8}$$

S5.5 Nuclear HP NEO-CCSD t^{en}-amplitude equation

$$0 = \langle \mu_\kappa^{\mathrm{en}} | \bar{H}_{\mathrm{NEO}} | 0^{\mathrm{e}} 0^{\mathrm{n}} \rangle$$

$$\begin{aligned}
0 = {} & (g^{\mathrm{en}}_\kappa)^{iI}_{aA} - (F^{\mathrm{e}})^{i}_{j}(t^{\mathrm{en}}_\kappa)^{jI}_{aA} + (F^{\mathrm{e}})^{b}_{a}(t^{\mathrm{en}}_\kappa)^{iI}_{bA} - (F^{\mathrm{n}}_\kappa)^{I}_{J}(t^{\mathrm{en}}_\kappa)^{iJ}_{aA} + (F^{\mathrm{n}}_\kappa)^{B}_{A}(t^{\mathrm{en}}_\kappa)^{iI}_{aB} - (g^{\mathrm{en}}_\kappa)^{iI}_{jA}(t^{\mathrm{e}})^{j}_{a} - \\
& (g^{\mathrm{en}}_\kappa)^{iI}_{aJ}(t^{\mathrm{n}}_\kappa)^{J}_{A} + (g^{\mathrm{en}}_\kappa)^{iB}_{aA}(t^{\mathrm{n}}_\kappa)^{I}_{B} + (g^{\mathrm{en}}_\kappa)^{bI}_{aA}(t^{\mathrm{e}})^{i}_{b} - (\bar{g}^{\mathrm{ee}})^{ib}_{ja}(t^{\mathrm{en}}_\kappa)^{jI}_{bA} + (g^{\mathrm{en}}_\kappa)^{iI}_{jJ}(t^{\mathrm{en}}_\kappa)^{jJ}_{aA} - (g^{\mathrm{en}}_\kappa)^{iB}_{jA}(t^{\mathrm{en}}_\kappa)^{jI}_{aB} - \\
& (g^{\mathrm{en}}_\kappa)^{bI}_{jA}(t^{\mathrm{ee}})^{ji}_{ab} - (g^{\mathrm{en}}_\kappa)^{bI}_{aJ}(t^{\mathrm{en}}_\kappa)^{iJ}_{bA} + (g^{\mathrm{en}}_\kappa)^{bB}_{aA}(t^{\mathrm{en}}_\kappa)^{iI}_{bB} - (F^{\mathrm{e}})^{b}_{j}(t^{\mathrm{e}})^{j}_{a}(t^{\mathrm{en}}_\kappa)^{iI}_{bA} - (F^{\mathrm{e}})^{b}_{j}(t^{\mathrm{e}})^{i}_{b}(t^{\mathrm{en}}_\kappa)^{jI}_{aA} - \\
& (F^{\mathrm{n}}_\kappa)^{B}_{J}(t^{\mathrm{n}}_\kappa)^{J}_{A}(t^{\mathrm{en}}_\kappa)^{iI}_{aB} - (F^{\mathrm{n}}_\kappa)^{B}_{J}(t^{\mathrm{n}}_\kappa)^{I}_{B}(t^{\mathrm{en}}_\kappa)^{iJ}_{aA} + (g^{\mathrm{en}}_\kappa)^{iI}_{jJ}(t^{\mathrm{e}})^{j}_{a}(t^{\mathrm{n}}_\kappa)^{J}_{A} - (g^{\mathrm{en}}_\kappa)^{iB}_{jA}(t^{\mathrm{e}})^{j}_{a}(t^{\mathrm{n}}_\kappa)^{I}_{B} - (g^{\mathrm{en}}_\kappa)^{bI}_{jA}(t^{\mathrm{e}})^{i}_{b}(t^{\mathrm{e}})^{j}_{a} - \\
& (g^{\mathrm{en}}_\kappa)^{iB}_{aJ}(t^{\mathrm{n}}_\kappa)^{I}_{B}(t^{\mathrm{n}}_\kappa)^{J}_{A} - (g^{\mathrm{en}}_\kappa)^{bI}_{aJ}(t^{\mathrm{e}})^{i}_{b}(t^{\mathrm{n}}_\kappa)^{J}_{A} + (g^{\mathrm{en}}_\kappa)^{bB}_{aA}(t^{\mathrm{e}})^{i}_{b}(t^{\mathrm{n}}_\kappa)^{I}_{B} - (\bar{g}^{\mathrm{ee}})^{ib}_{jk}(t^{\mathrm{e}})^{j}_{a}(t^{\mathrm{en}}_\kappa)^{kI}_{bA} + (\bar{g}^{\mathrm{ee}})^{ib}_{jk}(t^{\mathrm{e}})^{j}_{b}(t^{\mathrm{en}}_\kappa)^{kI}_{aA} + \\
& (\bar{g}^{\mathrm{ee}})^{bc}_{ja}(t^{\mathrm{e}})^{i}_{c}(t^{\mathrm{en}}_\kappa)^{jI}_{bA} - (\bar{g}^{\mathrm{ee}})^{bc}_{ja}(t^{\mathrm{e}})^{j}_{c}(t^{\mathrm{en}}_\kappa)^{iI}_{bA} + (g^{\mathrm{en}}_\kappa)^{iB}_{jJ}(t^{\mathrm{n}}_\kappa)^{J}_{A}(t^{\mathrm{en}}_\kappa)^{jI}_{aB} + (g^{\mathrm{en}}_\kappa)^{iB}_{jJ}(t^{\mathrm{n}}_\kappa)^{I}_{B}(t^{\mathrm{en}}_\kappa)^{jJ}_{aA} - \\
& (g^{\mathrm{en}}_\kappa)^{iB}_{jJ}(t^{\mathrm{n}}_\kappa)^{J}_{B}(t^{\mathrm{en}}_\kappa)^{jI}_{aA} + (g^{\mathrm{en}}_\kappa)^{bI}_{jJ}(t^{\mathrm{e}})^{j}_{a}(t^{\mathrm{en}}_\kappa)^{iJ}_{bA} + (g^{\mathrm{en}}_\kappa)^{bI}_{jJ}(t^{\mathrm{e}})^{i}_{b}(t^{\mathrm{en}}_\kappa)^{jJ}_{aA} - (g^{\mathrm{en}}_\kappa)^{bI}_{jJ}(t^{\mathrm{e}})^{j}_{b}(t^{\mathrm{en}}_\kappa)^{iJ}_{aA} + \\
& (g^{\mathrm{en}}_\kappa)^{bI}_{jJ}(t^{\mathrm{n}}_\kappa)^{J}_{A}(t^{\mathrm{ee}})^{ji}_{ab} - (g^{\mathrm{en}}_\kappa)^{bB}_{jA}(t^{\mathrm{e}})^{j}_{a}(t^{\mathrm{en}}_\kappa)^{iI}_{bB} - (g^{\mathrm{en}}_\kappa)^{bB}_{jA}(t^{\mathrm{e}})^{i}_{b}(t^{\mathrm{en}}_\kappa)^{jI}_{aB} + (g^{\mathrm{en}}_\kappa)^{bB}_{jA}(t^{\mathrm{e}})^{j}_{b}(t^{\mathrm{en}}_\kappa)^{iI}_{aB} - \\
& (g^{\mathrm{en}}_\kappa)^{bB}_{jA}(t^{\mathrm{n}}_\kappa)^{I}_{B}(t^{\mathrm{ee}})^{ji}_{ab} - (g^{\mathrm{en}}_\kappa)^{bB}_{aJ}(t^{\mathrm{n}}_\kappa)^{J}_{A}(t^{\mathrm{en}}_\kappa)^{iI}_{bB} - (g^{\mathrm{en}}_\kappa)^{bB}_{aJ}(t^{\mathrm{n}}_\kappa)^{I}_{B}(t^{\mathrm{en}}_\kappa)^{iJ}_{bA} + (g^{\mathrm{en}}_\kappa)^{bB}_{aJ}(t^{\mathrm{n}}_\kappa)^{J}_{B}(t^{\mathrm{en}}_\kappa)^{iI}_{bA} + \\
& (\bar{g}^{\mathrm{ee}})^{bc}_{jk}(t^{\mathrm{ee}})^{ji}_{ac}(t^{\mathrm{en}}_\kappa)^{kI}_{bA} + \tfrac{1}{2}(\bar{g}^{\mathrm{ee}})^{bc}_{jk}(t^{\mathrm{ee}})^{kj}_{ac}(t^{\mathrm{en}}_\kappa)^{iI}_{bA} + \tfrac{1}{2}(\bar{g}^{\mathrm{ee}})^{bc}_{jk}(t^{\mathrm{ee}})^{ji}_{cb}(t^{\mathrm{en}}_\kappa)^{kI}_{aA} - (g^{\mathrm{en}}_\kappa)^{bB}_{jJ}(t^{\mathrm{en}}_\kappa)^{iJ}_{aA}(t^{\mathrm{en}}_\kappa)^{jI}_{bB} - \\
& (g^{\mathrm{en}}_\kappa)^{bB}_{jJ}(t^{\mathrm{en}}_\kappa)^{iI}_{aB}(t^{\mathrm{en}}_\kappa)^{jJ}_{bA} + (g^{\mathrm{en}}_\kappa)^{bB}_{jJ}(t^{\mathrm{en}}_\kappa)^{iJ}_{aB}(t^{\mathrm{en}}_\kappa)^{jI}_{bA} - (g^{\mathrm{en}}_\kappa)^{bB}_{jJ}(t^{\mathrm{en}}_\kappa)^{iI}_{bA}(t^{\mathrm{en}}_\kappa)^{jJ}_{aB} + (g^{\mathrm{en}}_\kappa)^{bB}_{jJ}(t^{\mathrm{en}}_\kappa)^{iJ}_{bA}(t^{\mathrm{en}}_\kappa)^{jI}_{aB} + \\
& (g^{\mathrm{en}}_\kappa)^{bB}_{jJ}(t^{\mathrm{en}}_\kappa)^{iI}_{bB}(t^{\mathrm{en}}_\kappa)^{jJ}_{aA} - (g^{\mathrm{en}}_\kappa)^{bB}_{jJ}(t^{\mathrm{en}}_\kappa)^{iJ}_{bB}(t^{\mathrm{en}}_\kappa)^{jI}_{aA} + (g^{\mathrm{en}}_\kappa)^{iB}_{jJ}(t^{\mathrm{e}})^{j}_{a}(t^{\mathrm{n}}_\kappa)^{I}_{B}(t^{\mathrm{n}}_\kappa)^{J}_{A} + (g^{\mathrm{en}}_\kappa)^{bI}_{jJ}(t^{\mathrm{e}})^{i}_{b}(t^{\mathrm{e}})^{j}_{a}(t^{\mathrm{n}}_\kappa)^{J}_{A} - \\
& (g^{\mathrm{en}}_\kappa)^{bB}_{jA}(t^{\mathrm{e}})^{i}_{b}(t^{\mathrm{e}})^{j}_{a}(t^{\mathrm{n}}_\kappa)^{I}_{B} - (g^{\mathrm{en}}_\kappa)^{bB}_{aJ}(t^{\mathrm{e}})^{i}_{b}(t^{\mathrm{n}}_\kappa)^{I}_{B}(t^{\mathrm{n}}_\kappa)^{J}_{A} - (\bar{g}^{\mathrm{ee}})^{bc}_{jk}(t^{\mathrm{e}})^{j}_{a}(t^{\mathrm{e}})^{k}_{c}(t^{\mathrm{en}}_\kappa)^{iI}_{bA} + (\bar{g}^{\mathrm{ee}})^{bc}_{jk}(t^{\mathrm{e}})^{i}_{c}(t^{\mathrm{e}})^{j}_{a}(t^{\mathrm{en}}_\kappa)^{kI}_{bA} - \\
& (\bar{g}^{\mathrm{ee}})^{bc}_{jk}(t^{\mathrm{e}})^{i}_{c}(t^{\mathrm{e}})^{j}_{b}(t^{\mathrm{en}}_\kappa)^{kI}_{aA} + (g^{\mathrm{en}}_\kappa)^{bB}_{jJ}(t^{\mathrm{e}})^{j}_{a}(t^{\mathrm{n}}_\kappa)^{J}_{A}(t^{\mathrm{en}}_\kappa)^{iI}_{bB} + (g^{\mathrm{en}}_\kappa)^{bB}_{jJ}(t^{\mathrm{e}})^{j}_{a}(t^{\mathrm{n}}_\kappa)^{I}_{B}(t^{\mathrm{en}}_\kappa)^{iJ}_{bA} - \\
& (g^{\mathrm{en}}_\kappa)^{bB}_{jJ}(t^{\mathrm{e}})^{j}_{a}(t^{\mathrm{n}}_\kappa)^{J}_{B}(t^{\mathrm{en}}_\kappa)^{iI}_{bA} + (g^{\mathrm{en}}_\kappa)^{bB}_{jJ}(t^{\mathrm{e}})^{i}_{b}(t^{\mathrm{n}}_\kappa)^{J}_{A}(t^{\mathrm{en}}_\kappa)^{jI}_{aB} + (g^{\mathrm{en}}_\kappa)^{bB}_{jJ}(t^{\mathrm{e}})^{i}_{b}(t^{\mathrm{n}}_\kappa)^{I}_{B}(t^{\mathrm{en}}_\kappa)^{jJ}_{aA} - \\
& (g^{\mathrm{en}}_\kappa)^{bB}_{jJ}(t^{\mathrm{e}})^{i}_{b}(t^{\mathrm{n}}_\kappa)^{J}_{B}(t^{\mathrm{en}}_\kappa)^{jI}_{aA} - (g^{\mathrm{en}}_\kappa)^{bB}_{jJ}(t^{\mathrm{e}})^{j}_{b}(t^{\mathrm{n}}_\kappa)^{J}_{A}(t^{\mathrm{en}}_\kappa)^{iI}_{aB} - (g^{\mathrm{en}}_\kappa)^{bB}_{jJ}(t^{\mathrm{e}})^{j}_{b}(t^{\mathrm{n}}_\kappa)^{I}_{B}(t^{\mathrm{en}}_\kappa)^{iJ}_{aA} + \\
& (g^{\mathrm{en}}_\kappa)^{bB}_{jJ}(t^{\mathrm{n}}_\kappa)^{I}_{B}(t^{\mathrm{n}}_\kappa)^{J}_{A}(t^{\mathrm{ee}})^{ji}_{ab} + (g^{\mathrm{en}}_\kappa)^{bB}_{jJ}(t^{\mathrm{e}})^{i}_{b}(t^{\mathrm{e}})^{j}_{a}(t^{\mathrm{n}}_\kappa)^{I}_{B}(t^{\mathrm{n}}_\kappa)^{J}_{A} + \textstyle\sum_{\xi \in \mathcal{N}_{\mathrm{r}}} \big[(g^{\mathrm{nn}}_{\kappa\xi})^{IE}_{AM}(t^{\mathrm{en}}_\xi)^{iM}_{aE} - \\
& (g^{\mathrm{en}}_\xi)^{iE}_{jM}(t^{\mathrm{n}}_\xi)^{M}_{E}(t^{\mathrm{en}}_\kappa)^{jI}_{aA} + (g^{\mathrm{en}}_\xi)^{bE}_{aM}(t^{\mathrm{n}}_\xi)^{M}_{E}(t^{\mathrm{en}}_\kappa)^{iI}_{bA} - (g^{\mathrm{nn}}_{\kappa\xi})^{IE}_{JM}(t^{\mathrm{n}}_\kappa)^{J}_{A}(t^{\mathrm{en}}_\xi)^{iM}_{aE} - (g^{\mathrm{nn}}_{\kappa\xi})^{IE}_{JM}(t^{\mathrm{n}}_\xi)^{M}_{E}(t^{\mathrm{en}}_\kappa)^{iJ}_{aA} + \\
& (g^{\mathrm{nn}}_{\kappa\xi})^{BE}_{AM}(t^{\mathrm{n}}_\kappa)^{I}_{B}(t^{\mathrm{en}}_\xi)^{iM}_{aE} + (g^{\mathrm{nn}}_{\kappa\xi})^{BE}_{AM}(t^{\mathrm{n}}_\xi)^{M}_{E}(t^{\mathrm{en}}_\kappa)^{iI}_{aB} - (g^{\mathrm{en}}_\xi)^{bE}_{jM}(t^{\mathrm{en}}_\kappa)^{jI}_{aA}(t^{\mathrm{en}}_\xi)^{iM}_{bE} - (g^{\mathrm{en}}_\xi)^{bE}_{jM}(t^{\mathrm{en}}_\kappa)^{iI}_{bA}(t^{\mathrm{en}}_\xi)^{jM}_{aE} + \\
& (g^{\mathrm{en}}_\xi)^{bE}_{jM}(t^{\mathrm{en}}_\kappa)^{jI}_{bA}(t^{\mathrm{en}}_\xi)^{iM}_{aE} - (g^{\mathrm{en}}_\xi)^{bE}_{jM}(t^{\mathrm{e}})^{j}_{a}(t^{\mathrm{n}}_\xi)^{M}_{E}(t^{\mathrm{en}}_\kappa)^{iI}_{bA} - (g^{\mathrm{en}}_\xi)^{bE}_{jM}(t^{\mathrm{e}})^{i}_{b}(t^{\mathrm{n}}_\xi)^{M}_{E}(t^{\mathrm{en}}_\kappa)^{jI}_{aA} - \\
& (g^{\mathrm{nn}}_{\kappa\xi})^{BE}_{JM}(t^{\mathrm{n}}_\kappa)^{J}_{A}(t^{\mathrm{n}}_\xi)^{M}_{E}(t^{\mathrm{en}}_\kappa)^{iI}_{aB} - (g^{\mathrm{nn}}_{\kappa\xi})^{BE}_{JM}(t^{\mathrm{n}}_\kappa)^{I}_{B}(t^{\mathrm{n}}_\kappa)^{J}_{A}(t^{\mathrm{en}}_\xi)^{iM}_{aE} - (g^{\mathrm{nn}}_{\kappa\xi})^{BE}_{JM}(t^{\mathrm{n}}_\kappa)^{I}_{B}(t^{\mathrm{n}}_\xi)^{M}_{E}(t^{\mathrm{en}}_\kappa)^{iJ}_{aA} \big]
\end{aligned} \tag{S9}$$

S6 Procedure for generating programmable equations when including double nuclear excitations

In this section, we will go through the same simple procedure described in the main text for obtaining programmable energy and t-amplitude equations, except that double nuclear excitations will be included. We will only go through the parts of the process that have changed or are new, and the (nn) superscript will be used to indicate operators that change when double nuclear excitations are included. We define the double nuclear cluster operator as:

$$\hat{T}_{\gamma\eta}^{\mathrm{nn}} = \sum_{I\in\mathcal{S}_{\gamma}^{\mathrm{n(o)}}} \sum_{A\in\mathcal{S}_{\gamma}^{\mathrm{n(v)}}} \sum_{M\in\mathcal{S}_{\eta}^{\mathrm{n(o)}}} \sum_{E\in\mathcal{S}_{\eta}^{\mathrm{n(v)}}} (t_{\gamma\eta}^{\mathrm{nn}})_{AE}^{IM} \tilde{a}_{IM}^{AE} \tag{S10}$$

Thus, the new cluster operator can be written as

$$\hat{T}_{\text{NEO-CCSD}}^{(\mathrm{nn})} = \hat{T}^{\mathrm{e}} + \hat{T}^{\mathrm{ee}} + \sum_{\gamma\in\mathcal{N}_{\mathrm{q}}} \hat{T}_{\gamma}^{\mathrm{n}} + \sum_{\gamma\in\mathcal{N}_{\mathrm{q}}} \hat{T}_{\gamma}^{\mathrm{en}} + \sum_{\substack{\gamma,\eta\in\mathcal{N}_{\mathrm{q}}\\ \gamma<\eta}} \hat{T}_{\gamma\eta}^{\mathrm{nn}} \tag{S11}$$

The double nuclear t-amplitudes can be found through the following equation:

$$0 = \langle \mu_{\kappa\nu}^{\mathrm{nn}} | \bar{H}_{\mathrm{NEO}}^{(\mathrm{nn})} | 0^{\mathrm{e}} 0^{\mathrm{n}} \rangle \tag{S12}$$

where $\langle \mu_{\kappa\nu}^{\mathrm{nn}} | = \langle 0^{\mathrm{e}} 0^{\mathrm{n}} | \, a_{AE}^{IM}$ for $I \in \mathcal{S}_{\kappa}^{\mathrm{n(o)}}$, $M \in \mathcal{S}_{\nu}^{\mathrm{n(o)}}$, $A \in \mathcal{S}_{\kappa}^{\mathrm{n(v)}}$, and $E \in \mathcal{S}_{\nu}^{\mathrm{n(v)}}$. Moreover, $\bar{H}_{\mathrm{NEO}}^{(\mathrm{nn})} = e^{-\hat{T}_{\text{NEO-CCSD}}^{(\mathrm{nn})}} \hat{H}_{\mathrm{NEO}} e^{\hat{T}_{\text{NEO-CCSD}}^{(\mathrm{nn})}}$. Eq. (S12) must be solved for each distinct pair of quantum nuclei κ and ν. Eqs. (11a)–(11e) must also be modified to use $\bar{H}_{\mathrm{NEO}}^{(\mathrm{nn})}$ instead of $\bar{H}_{\mathrm{NEO}}$.

Because double nuclear excitations are included, when obtaining programmable equations for Eqs. (11a)–(11c), the representative cluster operator, $\hat{T}_{\text{NEO-CCSD}}^{\prime(\mathrm{nn})}$, must include double

nuclear excitations:

$$\hat{T}'^{(\mathrm{nn})}_{\text{NEO-CCSD}} = \hat{T}^{\mathrm{e}} + \hat{T}^{\mathrm{ee}} + \hat{T}^{\mathrm{n}}_{\gamma} + \hat{T}^{\mathrm{n}}_{\eta} + \hat{T}^{\mathrm{en}}_{\gamma} + \hat{T}^{\mathrm{en}}_{\eta} + \hat{T}^{\mathrm{nn}}_{\gamma\eta} \tag{S13}$$

When obtaining programmable equations for Eqs. (11d) and (11e), we must also include double nuclear excitations in the representative cluster operator, $\hat{T}''^{(\mathrm{nn})}_{\text{NEO-CCSD}}$. Furthermore, we must retain the term that was previously excluded when double nuclear excitations were not present. Thus, for these equations, the representative Hamiltonian, $\hat{H}''^{(\mathrm{nn})}_{\mathrm{NEO}}$, and the associated representative cluster operator, $\hat{T}''^{(\mathrm{nn})}_{\text{NEO-CCSD}}$, both involve three nuclei, κ, ξ, and η and can be written as

$$\hat{H}''^{(\mathrm{nn})}_{\mathrm{NEO}} = \hat{F}^{\mathrm{e}} + \hat{g}^{\mathrm{ee}} + \hat{F}^{\mathrm{n}}_{\kappa} + \hat{F}^{\mathrm{n}}_{\xi} + \hat{g}^{\mathrm{nn}}_{\kappa\xi} + \hat{g}^{\mathrm{nn}}_{\xi\eta} + \hat{g}^{\mathrm{en}}_{\kappa} + \hat{g}^{\mathrm{en}}_{\xi} \tag{S14a}$$

$$\begin{aligned}\hat{T}''^{(\mathrm{nn})}_{\text{NEO-CCSD}} = {} & \hat{T}^{\mathrm{e}} + \hat{T}^{\mathrm{ee}} + \hat{T}^{\mathrm{n}}_{\kappa} + \hat{T}^{\mathrm{n}}_{\xi} + \hat{T}^{\mathrm{n}}_{\eta} + \hat{T}^{\mathrm{en}}_{\kappa} + \hat{T}^{\mathrm{en}}_{\xi} + \hat{T}^{\mathrm{en}}_{\eta} \\ & + \hat{T}^{\mathrm{nn}}_{\kappa\xi} + \hat{T}^{\mathrm{nn}}_{\kappa\eta} + \hat{T}^{\mathrm{nn}}_{\xi\eta}\end{aligned} \tag{S14b}$$

Then, after applying Wick's theorem, in addition to the summations described in the main text, for any term that involves both the ξ and η nuclei, a summation $\sum_{\substack{\xi,\eta\in\mathcal{N}_{\mathrm{r}} \\ \xi<\eta}}$ is used.

Finally, we go through the procedure to obtain programmable equations for Eq. (S12). Since this equation contains a bra with a double nuclear excitation for nucleus κ and nucleus ν, we rewrite the Hamiltonian in relation to these nuclei explicitly using the set of remaining nuclei, $\mathcal{N}'_{\mathrm{r}} = \mathcal{N}_{\mathrm{q}} \setminus \{\kappa, \nu\}$, as follows:

$$\begin{aligned}\hat{H}_{\mathrm{NEO}} = {} & \hat{F}^{\mathrm{e}} + \hat{g}^{\mathrm{ee}} + \hat{F}^{\mathrm{n}}_{\kappa} + \hat{F}^{\mathrm{n}}_{\nu} + \sum_{\lambda\in\mathcal{N}'_{\mathrm{r}}} \hat{F}^{\mathrm{n}}_{\lambda} \\ & + \hat{g}^{\mathrm{nn}}_{\kappa\nu} + \sum_{\lambda\in\mathcal{N}'_{\mathrm{r}}} \hat{g}^{\mathrm{nn}}_{\kappa\lambda} + \sum_{\lambda\in\mathcal{N}'_{\mathrm{r}}} \hat{g}^{\mathrm{nn}}_{\nu\lambda} + \sum_{\substack{\lambda,\sigma\in\mathcal{N}'_{\mathrm{r}} \\ \lambda<\sigma}} \hat{g}^{\mathrm{nn}}_{\lambda\sigma} \\ & + \hat{g}^{\mathrm{en}}_{\kappa} + \hat{g}^{\mathrm{en}}_{\nu} + \sum_{\lambda\in\mathcal{N}'_{\mathrm{r}}} \hat{g}^{\mathrm{en}}_{\lambda}\end{aligned} \tag{S15}$$

Thus, the representative Hamiltonian, $\hat{H}_{\text{NEO}}^{\prime\prime\prime(\text{nn})}$, which selects one term from each summation in Eq. (S15), and the associated representative cluster operator, $\hat{T}_{\text{NEO-CCSD}}^{\prime\prime\prime(\text{nn})}$, both involve four nuclei and can be written as

$$
\begin{aligned}
\hat{H}_{\text{NEO}}^{\prime\prime\prime(\text{nn})} &= \hat{F}^{\text{e}} + \hat{g}^{\text{ee}} + \hat{F}_{\kappa}^{\text{n}} + \hat{F}_{\nu}^{\text{n}} + \hat{F}_{\lambda}^{\text{n}} \\
&+ \hat{g}_{\kappa\nu}^{\text{nn}} + \hat{g}_{\kappa\lambda}^{\text{nn}} + \hat{g}_{\nu\lambda}^{\text{nn}} + \hat{g}_{\lambda\sigma}^{\text{nn}} \\
&+ \hat{g}_{\kappa}^{\text{en}} + \hat{g}_{\nu}^{\text{en}} + \hat{g}_{\lambda}^{\text{en}} \qquad \text{(S16)} \\
\hat{T}_{\text{NEO-CCSD}}^{\prime\prime\prime(\text{nn})} &= \hat{T}^{\text{e}} + \hat{T}^{\text{ee}} + \hat{T}_{\kappa}^{\text{n}} + \hat{T}_{\nu}^{\text{n}} + \hat{T}_{\lambda}^{\text{n}} + \hat{T}_{\sigma}^{\text{n}} \\
&+ \hat{T}_{\kappa}^{\text{en}} + \hat{T}_{\nu}^{\text{en}} + \hat{T}_{\lambda}^{\text{en}} + \hat{T}_{\sigma}^{\text{en}} \\
&+ \hat{T}_{\kappa\nu}^{\text{nn}} + \hat{T}_{\kappa\lambda}^{\text{nn}} + \hat{T}_{\kappa\sigma}^{\text{nn}} + \hat{T}_{\nu\lambda}^{\text{nn}} + \hat{T}_{\nu\sigma}^{\text{nn}} + \hat{T}_{\lambda\sigma}^{\text{nn}} \qquad \text{(S17)}
\end{aligned}
$$

Wick's theorem can be applied to Eq. (S12) using Eqs. (S16) and (S17) to obtain programmable equations because only four occupied and four virtual nuclear spaces need to be considered. Then, in the programmable equations for any term involving the λ nucleus, a summation $\sum_{\lambda \in \mathcal{N}_{\text{r}}'}$ is used. For any term involving both the λ and the σ nuclei, a summation $\sum_{\substack{\lambda,\sigma \in \mathcal{N}_{\text{r}}' \\ \lambda < \sigma}}$ is used.

References

(1) Chow, M.; Paenurk, E.; Hammes-Schiffer, S. Efficient and robust optimization of nuclear and electronic orbitals within the nuclear Hartree product representation. *J. Chem. Phys.* **2026**, *164*, 074103.

(2) Pavošević, F.; Tao, Z.; Hammes-Schiffer, S. Multicomponent Coupled Cluster Singles and Doubles with Density Fitting: Protonated Water Tetramers with Quantized Protons. *J. Phys. Chem. Lett.* **2021**, *12*, 1631–1637.
